# Supervised AI and Deep Neural Networks to Evaluate High-Entropy Alloys as Reduction Catalysts in Aqueous Environments

**DOI:** 10.1021/acscatal.3c05017

**Published:** 2024-02-22

**Authors:** Rafael B. Araujo, Tomas Edvinsson

**Affiliations:** † Department of Materials Science and Engineering, Solid State Physics, Uppsala University, Box 35, 75103 Uppsala, Sweden; ‡ Energy Materials Laboratory, School of Natural and Environmental Science, Newcastle University, NE1 7RU Newcastle Upon Tyne, U.K.

**Keywords:** machine learning, deep neural networks, high-entropy alloys, scaling relations, competitive data analysis, DFT

## Abstract

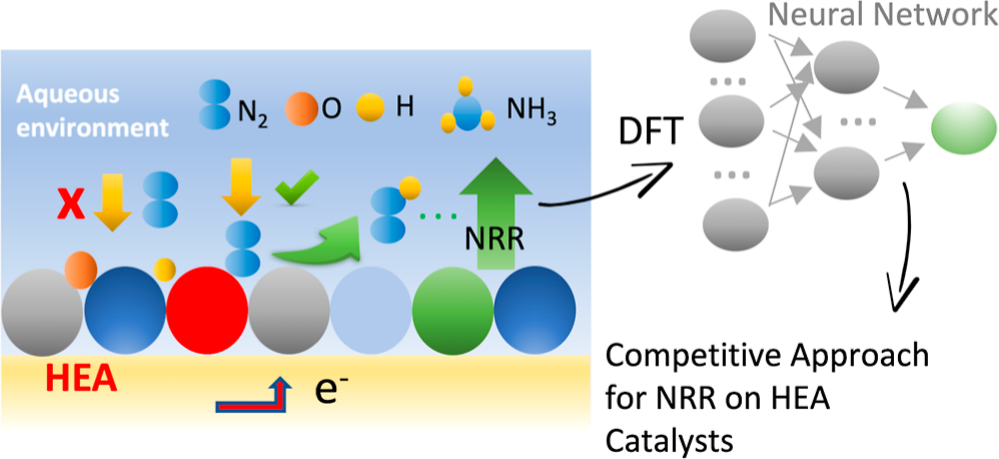

Competitive surface adsorption energies on catalytic surfaces constitute a fundamental aspect of modeling electrochemical reactions in aqueous environments. The conventional approach to this task relies on applying density functional theory, albeit with computationally intensive demands, particularly when dealing with intricate surfaces. In this study, we present a methodological exposition of quantifying competitive relationships within complex systems. Our methodology leverages quantum-mechanical-guided deep neural networks, deployed in the investigation of quinary high-entropy alloys composed of Mo–Cr–Mn–Fe–Co–Ni–Cu–Zn. These alloys are under examination as prospective electrocatalysts, facilitating the electrochemical synthesis of ammonia in aqueous media. Even in the most favorable scenario for nitrogen fixation identified in this study, at the transition from O and OH coverage to surface hydrogenation, the probability of N_2_ coverage remains low. This underscores the fact that catalyst optimization alone is insufficient for achieving efficient nitrogen reduction. In particular, these insights illuminate that system consideration with oxygen- and hydrogen-repelling approaches or high-pressure solutions would be necessary for improved nitrogen reduction within an aqueous environment.

## 1 Introduction

In recent years, artificial intelligence (AI) has emerged as a pivotal player in materials development. One particularly intriguing facet of this development is the utilization of AI models to forecast the emergence of novel energy-related materials, a trend that has garnered significant attention in the scientific community.^1−3^ This trend is poised to further intensify, propelled by the continuous expansion of data sets that encompass, for instance, quantum-mechanical calculations generated under well-defined theoretical frameworks, as well as the ongoing refinement and sophistication of AI algorithms. Within this context, the realm of catalysis-related applications stands out as a prime beneficiary of the synergy between AI algorithms and quantum-mechanical calculation data sets.^4,5^ Primarily, AI algorithms have adopted descriptor-based methodologies, which enable the quantification of material properties. These quantified properties, in turn, serve as critical inputs for high-throughput screening endeavors.^2^ This methodology has significantly streamlined the computational efforts required to identify materials with specific, desired properties, thereby affording researchers the opportunity to explore materials with increased complexity and intricacy.^6^

Ammonia (NH_3_) is essential to the global economy as a fertilizer feedstock and industrial chemical. Moreover, NH_3_ emerges as a potential energy vector that benefits from its high hydrogen content and easy liquefaction. For over a hundred years, the primary approach to produce NH_3_, named Haber-Bosch, stands at the heating of pressurized N_2_ and H_2_ molecules and, further, catalyzed over a Fe-based catalyst to form NH_3_. This approach emits CO_2_ into the atmosphere once it is based on hydrogen obtained by steam reforming, enhancing global warming. In this context, the electrocatalytic reduction of N_2_ powered by clean energy sources, e.g., solar or wind, is a sustainable alternative to Haber-Bosch. Electrochemical NH_3_ production can be performed at 100 °C and atmospheric pressure, thus requiring much fewer investments and allowing a distributed production over countries with less or no infrastructure. However, the implementation of this technology has been hindered by the low efficiency, typically a few percent, of the currently used catalysts that, among other things, is due to the competition between NRR and hydrogen evolution reaction (HER) together with the N_2_ fixation issue.

The concept of multicomponent alloys with entropy stabilization came out around 2004 when two independent research groups showed that multiple-element materials containing at least five different species could be formed in a homogeneous phase.^7,8^ The thermodynamically and kinetically stabilized structure of high-entropy alloys provides high fracture resistance, ductility, and physicochemical stability, thus enabling them to function under harsh environments.^9−11^ The formation of a single stable uniform phase or multiphase state in high-entropy alloys can be accessed via the Gibbs free-energy equation: Δ*G*_mix_ = Δ*H*_mix_ – *T* × Δ*S*_mix_ where, Δ*G*_mix_, Δ*H*_mix_ and Δ*S*_mix_ are the changes of the Gibbs free energy, mixing enthalpy, and configurational entropy, respectively, and *T* is the temperature. Once Δ*G*_mix_ presents lower energy than the phase separated phases, a solid solution is then expected. High-entropy materials (HEM) are disordered multicomponent structures stabilized via maximization of the configurational entropy term of the Gibbs free energy variation. The configurational entropy can be expressed as, Δ*S*_mix_ = −*R*∑*C*_*i*_ ln *C*_*i*_, where *C*_*i*_ is the concentration of the *i*th component of the HEM. Therefore, two main aspects are essential for maximizing the configurational entropic effects and producing a single-phase solution: (i) sufficient high temperatures and (ii) incorporating several elements in the material with similar molar fractions during mixing. A minimum of five elements is typically required to obtain sufficiently high values of Δ*S*_mix_ and to provide a stabilized single-phase HEM.

Here, quinary HEAs made of earth-abundant elements Mo–Cr–Mn–Fe–Co–Ni–Cu–Zn are investigated as potential electrocatalysts for nitrogen reduction reaction in aqueous environment. Addressing the wide range of compositional space found in the quinary HEAs toward alternative catalysts for NRR is a challenging task that is rationalized here by employing density functional theory (DFT), deep neural networks, a probabilistic approach and also correlations with the HEAs intrinsic properties. A similar strategy has already been reported to optimize HEAs, in general,^12−14^ or specifically for CO_2_ reduction (experimentally confirmed),^15^ oxygen reduction reaction,^16^ NH_3_ decomposition,^17^ and NH_3_ production.^18^ The latter is being investigated by us but considering a gas diffusion electrode. We are here extending this strategy to seek NNR-efficient HEA catalysts in an aqueous environment.

The first difficulty in achieving an efficient NRR comes with the inertness of the N_2_ molecules, which leads to low N_2_ surface coverages. Indeed, in an alkaline environment, it is likely that catalytic surfaces will be oxidized, with hydroxyl groups, or hydrogenated under reductive conditions, depending on the applied potential. Therefore, N_2_ coverages compete with the adsorption of species like O*, OH*, and H*, which in turn dominate at differently applied potentials. We included this aspect in this investigation by modifying the probabilistic approach to consider competitive relations. In simple words, the probabilistic approach estimates the catalytic activity of a HEA by counting the active sites for NRR on the HEA surface. This is further employed to optimize catalyst elements and concentration. However, here, competitive relations are also accounted for in the probability of finding active sites for NRR on the HEA surface. For instance, only sites that have N_2_ adsorption stronger than O*, OH*, and H* are maximized, instead of accounting only the N_2_ adsorption in itself. This allows us to identify candidates that mitigate the difficulty of N_2_ fixation on the catalytic surfaces at a certain potential for an aqueous environment. Once N_2_ reaches the catalytic surface, the next challenge is to properly assess the chain of subsequent reactions, where this is approached by identifying the thermodynamical limiting steps of the NRR reaction, here set to N_2_* + H^+^ +e^–^ → NNH* and NH* + H^+^ + e^–^ → NHH*. Here, the probabilistic approach maximizes the sites where these reactions occur with a smaller thermodynamical step.

Turning to the employed approach to describe the referent electrochemical reaction, the work of Tayyebi et al.^19^ and Höskuldsson et al.^20^ have shown that including activation barriers in the calculation of N_2_ reduction pathways leads to the same electrochemical paths predicted with thermochemistry, for Ru(0001) and W(110). This also agrees with the works of Sharada et al.^21^ and Araujo et al.^22^ showing that transition states resemble final states for chemisorbed small molecules; hence, barriers are similar to thermodynamical steps. Thus, the computational hydrogen electrode method can be reliably used here.^23^ The probabilistic approach together with the computational hydrogen electrode and the machine learning technique permitted the analysis of 9668 HEAs for NH_3_ production and their merits for the reaction in aqueous media. The collected data are added to a database, and different scenarios are then discussed, where, for each case, a HEA is recommended as an alternative catalyst. Although the approach is applied to quantum-mechanical-guided selection of catalysts for nitrogen reduction, the scheme can be used for any competitive relationship, provided that a relevant evaluation function can be defined.

## 2 Method

**Figure 1:**
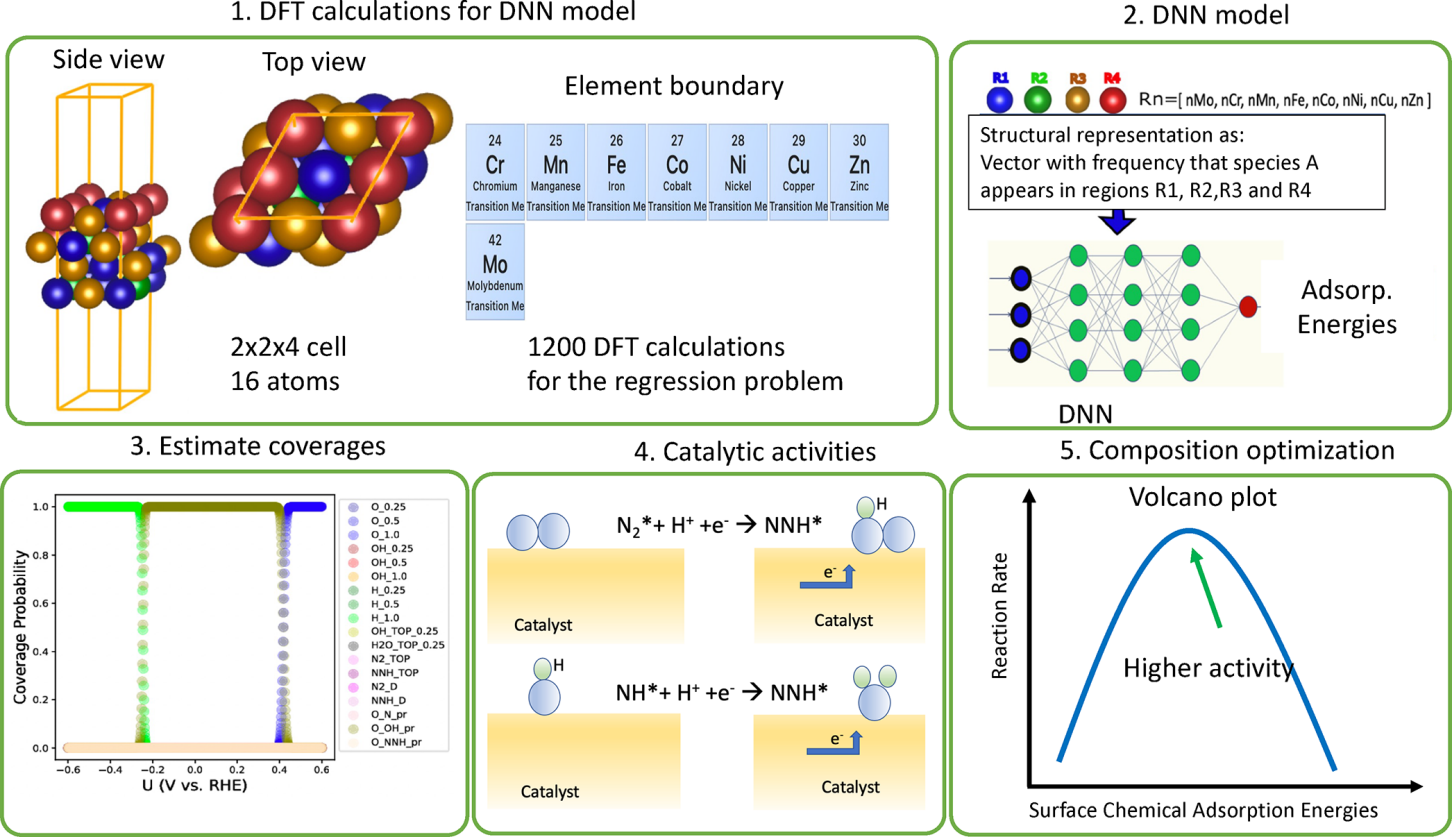
Schematic illustration of the steps to optimize HEA catalysts for NRR in a water medium. For the first step (1), the used structures and slab models for the DFT calculations are displayed together with the elemental composition. (2) Infrastructure to proceed with the neural network models are displayed. (3) Representation of the catalytic surface coverage is displayed. (4) Schematic representation of the two steps of the NRR considered the limiting thermodynamical steps. (5) Volcano-shaped plot representing how we will select the optimal elemental concentration of the HEA for high-performance NRR.

The approach employed to model the HEAs and estimate their activities toward NRR will be divided into different parts, as shown in Figure 1. In the first part, DFT calculations are performed over thousands of randomly created slabs that represent HEA surfaces formed with the elements Mo–Mn–Fe–Co–Ni–Cu–Zn. Representation models of the microstructures are created to establish a deep neural network (DNN) in the second part. This permits the computation of adsorption energies almost instantaneously, helping to circumvent the time-demanding DFT calculations (the most computationally time-demanding part in this scheme). In the third part, we quantify surface coverage probabilities at different applied potentials. This is important when considering an aqueous environment since coverages could poison the catalytic sites of the HEA surface and turn the catalyst inactive. The calculation of the coverages also estimates the potential where the probability of forming N_2_* is the highest. In the fourth step, a competing probabilistic approach calculates the HEA activity. Finally, the computed catalytic activities are correlated with the HEA’s intrinsic properties like the averaged valence electron concentration (VEC), averaged electronegativity (ELE—Pauling scale), and the averaged work function (WF) to rationalize the results in terms of their chemical properties.

It is known from different reports that the potential limiting step (PLS) dictating the activity of NRR is the first protonation of N_2_ forming NNH*, for catalysts formed mainly with later transition metals, or NH* + H^+^ + e^–^ → NHH*, for the case in which catalysts are formed with earlier transition metals.^19,20,24,25^ For the latter case, high energetic demands are needed to remove the strongly bonded NH* from the hollow threefold site (N forming three sigma bonds with the surface) and add it to the bridge site (two sigma bonds with the surface), as NHH*, hence, being the PLS. On the other hand, NH* does not adsorb strongly on later transition-metal surfaces (lower interaction since their d states are fully filled). However, N_2_ tends to adsorb vertically in later transition metals like that on Ru,^19^ hence, providing lower polarization of the N_2_ molecules that create difficulties to be activated. This is not the case for earlier transition metals like tungsten (W), since dinitrogen might likely sit horizontally on its catalytic surface. This allows for a greater polarization of the N_2_ molecules, facilitating the first hydrogenation step.^20^ The likeness in the bond mechanisms between the HEA catalytic surfaces and NRR intermediates when compared to transition metals can be anticipated, allowing us to presume similar PDSs for the NRR reaction. It is crucial to note, however, that deviations from this assumption may manifest at specific catalytic sites on the HEA surface. A comparable strategy was employed by Pedersen et al.^15^ in their exploration of HEA for CO_2_ and CO reduction. Their findings underwent further experimental validation, affirming the robustness of this statistical approach in comprehending the catalytic dynamics of HEA surfaces. With that in mind, the competitive probabilistic approach has estimated the catalytic activities by accounting for N_2_ adsorption versus the related counterparts, finding active sites for the PSL reactions, and relating to sites where H^+^ prefers to attach to N_2_, instead of going to the catalytic surface. These parameters are used to gauge the activity of different HEAs. The optimization of the best HEA is carried out by performing the previously discussed analysis for 9668 HEAs with five elements among Mo–Mn–Fe–Co–Ni–Cu–Zn. Ranks are determined based on two distinct scenarios that depend on the NRR reaction pathway.

### 2.1 DFT Calculations for the DNN Model

The first assumption of our model concerns the bond formed between small molecules and catalytic surfaces. A local character of the surface molecule bond mechanism is assumed, hence, determined by the microstructure of the local sites of the high-entropy alloy. This means that the vast composition of HEAs can be approached as an average over the microstructures of the referent HEA. The basic principles of the approach have been proposed by Batchelor et al.^16^ and further extended by Pedersen et al.^15^ to discover novel HEA catalysts for CO and CO_2_ reductions and, subsequently, experimentally validated. In this work, we deal with quinary HEAs containing elements among Mo–Cr–Mn–Fe–Co–Ni–Cu–Zn. Here, the microstructures of the HEAs for the regression task (DNN model) were built to cover all parts of the compositional space; hence, the models are trained over all possible adsorption energies delivered by such a combination of species that HEAs with five elements can provide. The computational hydrogen electrode approach, as proposed by Nørskov,^23^ was applied to model the electrochemical reactions. This approach assumes a coupled electron–proton transfer, simplifying the demanding calculation of solvation energies of ionic species. Here, the electrochemical/chemical transformation to calculate surface coverages and catalytic activities is
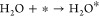
1
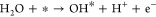
2
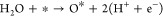
3
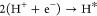
4
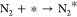
5
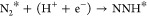
6
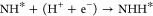
7The free-energy variation of each electrochemical/chemical reaction was calculated for 1200 microstructures as
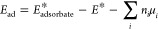
8where *E*_adsorbate_* is the self-consistent field (SCF) energy of the adsorbed intermediate corrected by the zero-point energy (ZPE) of the adsorbate and the solvation energies calculated with an implicit solvation method, E* is the SCF energy of the pure slab, and *n*_*i*_ is the number of species *i* with chemical potential μ_*i*_. ZPE and solvation energies of each intermediate adsorbed on the microstructures were computed for 10 cases and, then, used to correct the all-adsorption energies (values used are shown in Table S1). Moreover, μ_H_, μ_H_2_O_, μ_O_, and μ_N_ are the chemical potentials of hydrogen, water, oxygen, and nitrogen, respectively, that are obtained as
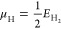
9
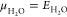
10
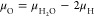
11
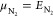
12
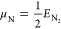
13
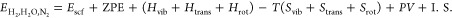
14Here, *E*_N_2__, *E*_H_2_O_ and *E*_H_2__ are the gas-phase Gibbs free energy computed as shown in eq 8, *E*_scf_ is the SCF energy, *H* refers to the enthalpic thermal contribution and *S* to the entropic thermal contribution. I.S. refers to the implicit solvation energy.

The electrochemical transformations have also accounted for distinct molecular configurations on the HEA surfaces. N_2_ molecules and NNH molecules were considered in vertical and horizontal adsorption configurations. Water molecules adsorb on the top site. It is known that water molecules tend to form an ice-like structure on the surface of catalysts like Pt.^26^ However, here, we have not added such a preassumption as the randomness of HEAs tends to break such water configuration, leading to a more disordered state. OH* adsorbs on the threefold HCP site and on the top site. O* and H* were accounted for on the threefold HCP sites. Adsorption configurations are summarized in Figure S1.

All adsorption energies were calculated using the projected augmented-wave method to solve the Kohn–Sham equations implemented in the Vienna ab initio Simulation Package (VASP).^27,28^ The wave functions were expanded using plane waves with a cutoff energy of 450 eV, while a (4 × 4 × 1) *k*-point mesh was used to sample over the Brillouin zone. A smearing of 0.2 eV was employed to obtain partial occupations using the Methfessel–Paxton scheme of second order. Spin-polarized orbitals were used in the ferromagnetic (FM) state, and the Bayesian error estimation functional with van der Waals correlation (BEEF-vdW)^29^ was utilized to describe the Kohn–Sham Hamiltonian exchange and correlation term. The BEEF-vdW has been reported to be one of the most accurate functionals to describe adsorption energies on transition-metal surfaces^30,31^ and is the approach chosen for this study. The structural models were built into a 2 × 2 × 4 face-centered cubic (FCC) (111) slab with a vacuum of 20 Å to avoid interaction among periodic images, allowing the two topmost layers to geometrically relax. In contrast, the two bottom layers were fixed to the optimized bulk structure. Atoms’ positions were optimized until a maximum force of 0.08 eV/Å was obtained. Lattice parameters of the slabs were set on a weighted average basis and assuming the species has FCC bulk structures, similar to the work of Batchelor et al.^16^ Moreover, Clausen et al.^32^ showed that the possible remaining strain effects on the adsorption energy of small molecules are alleviated by the inherent distortion of the lattice in HEAs. Bulk optimizations were performed with a *k*-point mesh of 15 × 15 × 15 in an FCC structure, and the obtained lattice parameters are summarized in Table S2. Vibrational modes were computed through the finite difference approximation, and solvation effects were calculated using a continuum solvation model developed by the Hennig group, as implemented in the VASPsol code.^33^ Moreover, the rotational, translational, and vibrational contributions to entropy and enthalpy were considered for gas-phase species where we furthermore set *PV* = *k*_B_*T* (see eq 6), where *P* and *V* are pressure and volume, respectively, while *T* and *k*_B_ are temperature and the Boltzmann constant, respectively.

Every computed adsorption energy was analyzed in terms of the total magnetic state of the used cell in the slab and slab + adsorbate. From the 1200 cases calculated for each adsorbate, we have removed the cases where the magnetization difference, slabs and slabs + adsorbate, is higher than 1μ_b_. The complexity of working with structures that include magnetic elements can lead to SCF convergences with very distinct magnetic states and coupling, hence, not representing the real interaction between the adsorbate and slab. Moreover, cases where the intermediates have moved from one adsorption site to another were also removed from the data set since those would not be representative to feed the deep neural network.

### 2.2 Deep Neural Networks

To circumvent the time-demanding DFT calculations, a representation model of the microstructures that enables establishing a deep neural network (DNN) model permitting the computation adsorption energies almost instantaneous was built. The representation used to feed the DNN involves the specification of four regions of the HEA microstructures and, hence, frequency counting of species on each specific region (Figure 1). These regions are then concatenated into a vector defining a regression problem, Δ*E*_N,N_2__ = ∑_*p*_^*R*^∑_*k*_^metals^*C*_*p*,*k*_*N*_*p*,*k*_^*i*^, where *N*_*p*,*k*_^*i*^ is the number of atoms of species *k* in the region *p* and *R* is the total number of regions, solved with the DNN. Each built vector represents one microstructure of a HEA of a specific concentration.

The DNNs were built using the Keras library.^34^ The data were trained in several networks where the best models were composed of dense sequential layers. The input layers were set with a linear activation function, while a “relu” activation function (L2 regularization function was employed in both cases) was used for the hidden layers. The output layers were built with a linear function. The loss function (mean-squared error, MSE) between the predicted adsorption energies and DFT-computed adsorption energies was minimized using an Adam optimizer. Our data set utilized to build the DNN was randomly divided into a training set (70%) and a test set (30%) for all adsorption energies. In general, the highest found MAE is 0.19 for the case N_2_ adsorbed horizontally (Figure S2). Others have reported ML-predicted MAEs of about 0.2 eV regarding the DFT adsorption energies,^35^ which inherently also have an error of about 0.2 eV within the Beef-vdW functional.^30,31^ Therefore, it pays off the employment of these models in pro of a considerable gain in computational time, allowing the removal of unpromising catalysts to be experimentally processed or by DFT calculations. Summary of the used DNN models are listed in the Supporting Information.

### 2.3 Surface Coverage

Surface coverages were computed using the DNN adsorption energies. For each HEA, 2000 microstructures of each adsorbate were calculated and used to build the Pourbaix-like diagrams. In this work, coverages are defined as the ratio between the number of adsorbates and the number of surface atoms. For the cases of H_2_O*, N_2_* (vertical and horizontal), NNH* (vertical and horizontal), and OH* on the top position, coverages of 0.25 ML were used to build the diagrams. For the cases of O*, OH*, and H*, coverages of 0.25, 0.5, and 1 ML were investigated. However, to adapt the calculations of coverages to the employed method, the effects of the coverages were added into an average scheme. This means that for coverages of 0.5 and 1 ML, adsorption energies of 0.25 ML were recomputed using DFT by adding extra adsorbates on the surface of 30 microstructures. Finally, the difference between the recomputed adsorption energies and the values with no extra adsorbate (difference between the cases of 0.25 and 0.5 ML, for instance) were added to the adsorption energies, as an effect of lateral interaction. In other words, the adsorption energies of the cases with 0.25 ML coverages are computed with the DNN. These values are, hence, modified by adding an average value referent to the lateral interaction for the cases of 0.5 and 1.0 ML coverages.

The probability of existence of each surface coverage was calculated as
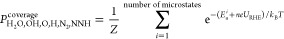
15
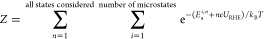
16Here, *U*_RHE_ is the potential measured against the reversible hydrogen electrode, *Z* is the partition function of the ensemble (normalization), *k*_B_ is the Boltzmann constant, *T* is the temperature and *ne* refers to the number of electrons participating in the electrochemical reaction. *U*_RHE_ has varied from −0.6 V vs RHE up to 0.6 V vs RHE. In other words, we compute the probability of a specific coverage for each value of *U*_RHE_. As already mentioned, coverage effects were added via an average approach where for O* coverages of 0.5 and 1 ML, 0.64 and 1.6 eV were added to the adsorption energies of the case 0.25 ML, respectively. For OH* coverages of 0.5 and 1 ML on the HPC hollow, 0.33 and 1.44 eV were added, respectively. For H*, 0.1 and 0.15 eV were added for 0.5 and 1 ML, respectively. The case of hydrogen is the one showing smaller lateral interaction. This indicates that full coverage of the catalytic surfaces will be more prone to exist than in cases where the surface still shows active sites.

### 2.4 Catalytic Activity

**Figure 2:**
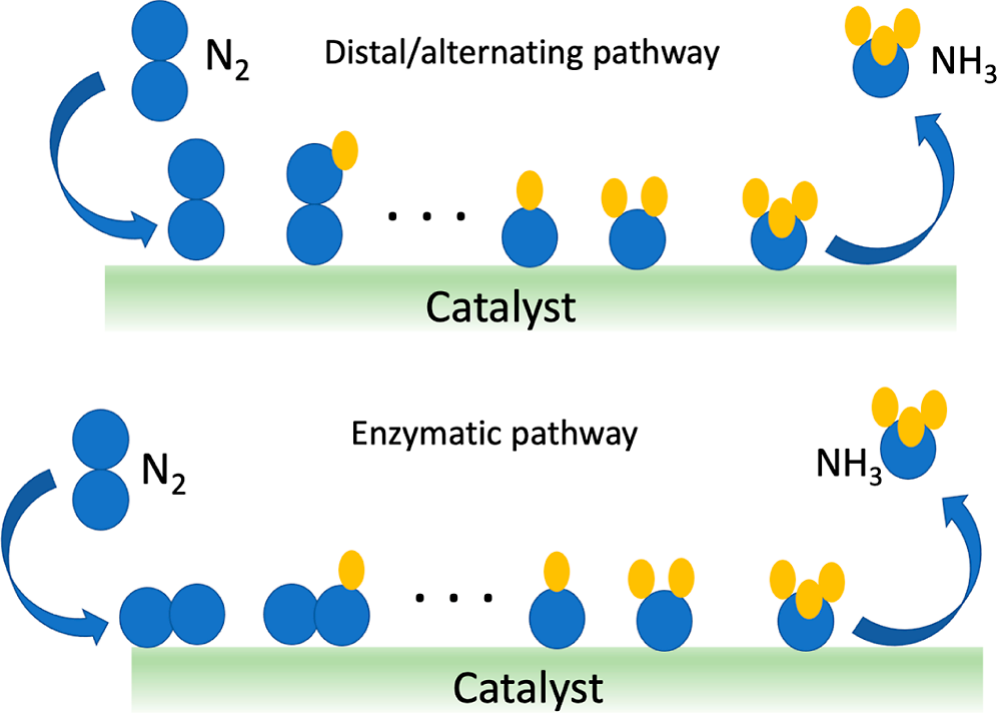
Schematic illustration of the reaction pathways for NRR: (a) distal/alternating; (b) enzymatic.

The catalytic activities (CA) are estimated here by employing a probabilistic approach.^16^ The computed CAs are divided into two cases: (i) the case where N_2_ adsorbs and the NNR follows an enzymatic pathway (Figure 2);^36^ (ii) the case where N_2_ adsorbs vertically and the reaction would likely proceed in the distal/alternating pathway (Figure 2).^36^ N_2_ reconfiguration from a vertical to a horizontal position is a nonelectrochemical process that displays a barrier of 0.60 eV when considering Ru(0001), as calculated by Tayyebi et al.,^19^ or 0.34 eV computed for W(110).^20^ Hence, by generalizing the reported results for Ru and W to other transition-metal catalysts, crossing between the two cases is unlikely. Moreover, we assume that NRR will occur via an H^+^ attachment of the adsorbed N_2_* forming NNH*—sequential proton–electron transfer.

CAs are, hence, computed for each HEA as follows:

I The electrochemical potential where N_2_ adsorption is more likely to exist is calculated (discussed in the Surface Coverage section). The free-energy variation of 2000 microstates for the reactions eqs 1–7 are computed at the referent potential. All cases among the 2000 microstructures where N_2_ adsorbs more exothermically than the competing species (H_2_O*, OH*, O*, and H*) are accounted to the probabilistic approach by summing them up and dividing by 2000. Hence, this estimates the competing probabilities of finding N_2_ adsorbed in the catalytic surface.
II We assume that the potential limiting steps of the reaction are N_2_* + H^+^ + e^–^ → NNH and NH* + H^+^ + e^–^ → NHH*. We calculate the free-energy variation of these reactions on the 2000 microstates of specific HAE at the value of *U*_RHE_ with the highest N_2_ coverage probability (as in the preview step). Exothermic cases are accounted for and then divided by 2000 to define the probability of finding active sites for these reactions, respectively.
III For the case of NRR proceeding via the enzymatic pathway, an extra parameter is added to balance the probability of H^+^ attacking the N_2_-adsorbed molecules. In this case, we compare the energetics of H^+^ going to (i) N_2_* to form NNH*, (ii) O* to form OH*, (ii) going to the pure surface at the applied potential where N_2_ adsorption is more likely. The cases where the formation of NNH* is preferred versus the others are accounted for and normalized by 2000 to deliver a competitive probability of forming NNH*. Unfortunately, this parameter cannot be accounted for in the case of the reaction going through the distal/alternating pathway due to technical reasons.
IV CAs for the different cases are calculated by multiplying the probabilities found in steps 1, 2, and 3 for N_2_ adsorbed horizontally (enzymatic pathway) and steps 1 and 2 for N_2_ adsorbed vertically.

### 2.5 Compositional Optimization

Composition optimization is performed by randomly creating 9668 HEAs formed of five elements among Mo–Mn–Fe–Co–Ni–Cu–Zn. CAs were computed as described previously for each case. These data were added to a database and ranks were determined.

## 3 Results

Results are divided into three main parts. In the first part, coverages are discussed together with the potentials where N_2_ coverage is more likely to exist. Two limit cases are, hence, closely evaluated. In the second part, the obtained CAs are presented for both the cases (enzymatic and distal alternating pathways), as computed for the 9668 quinary HEAs formed with the elements Mo–Mn–Fe–Co–Ni–Cu–Zn. The correlations with their intrinsic properties VEC, ELE, and WF and elemental concentrations are also analyzed. In the third part, the selected cases for both pathways (enzymatic and distal/alternation) are closely evaluated.

### 3.1 Coverages

**Figure 3:**
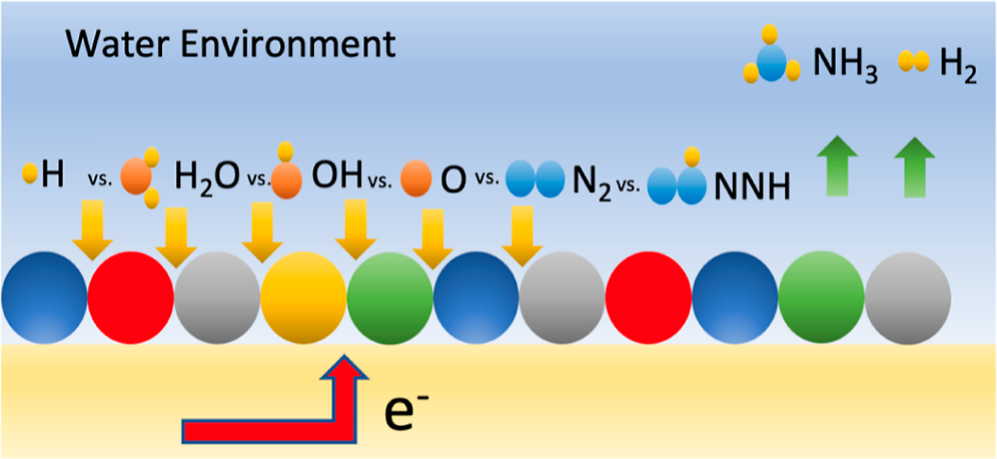
Schematic illustration of the competing coverages on a HEA catalytic surface in a water environment.

The surface coverages are investigated to gain insights into their probabilities under different applied potentials (potentials varying from −0.6 V vs RHE up to 0.6 V vs RHE). We account for surfaces covered by H_2_O*, OH*, O*, H*, N_2_*, and NNH* on their distinct adsorption sites (Figure 3). Moreover, for O*, OH*, and H*, coverages of 0.25, 0.5, and 1 ML are investigated, while for the other cases, N_2_*, NNH*, OH* on the top, and H_2_O*, only 0.25 ML coverage is used.

**Figure 4:**
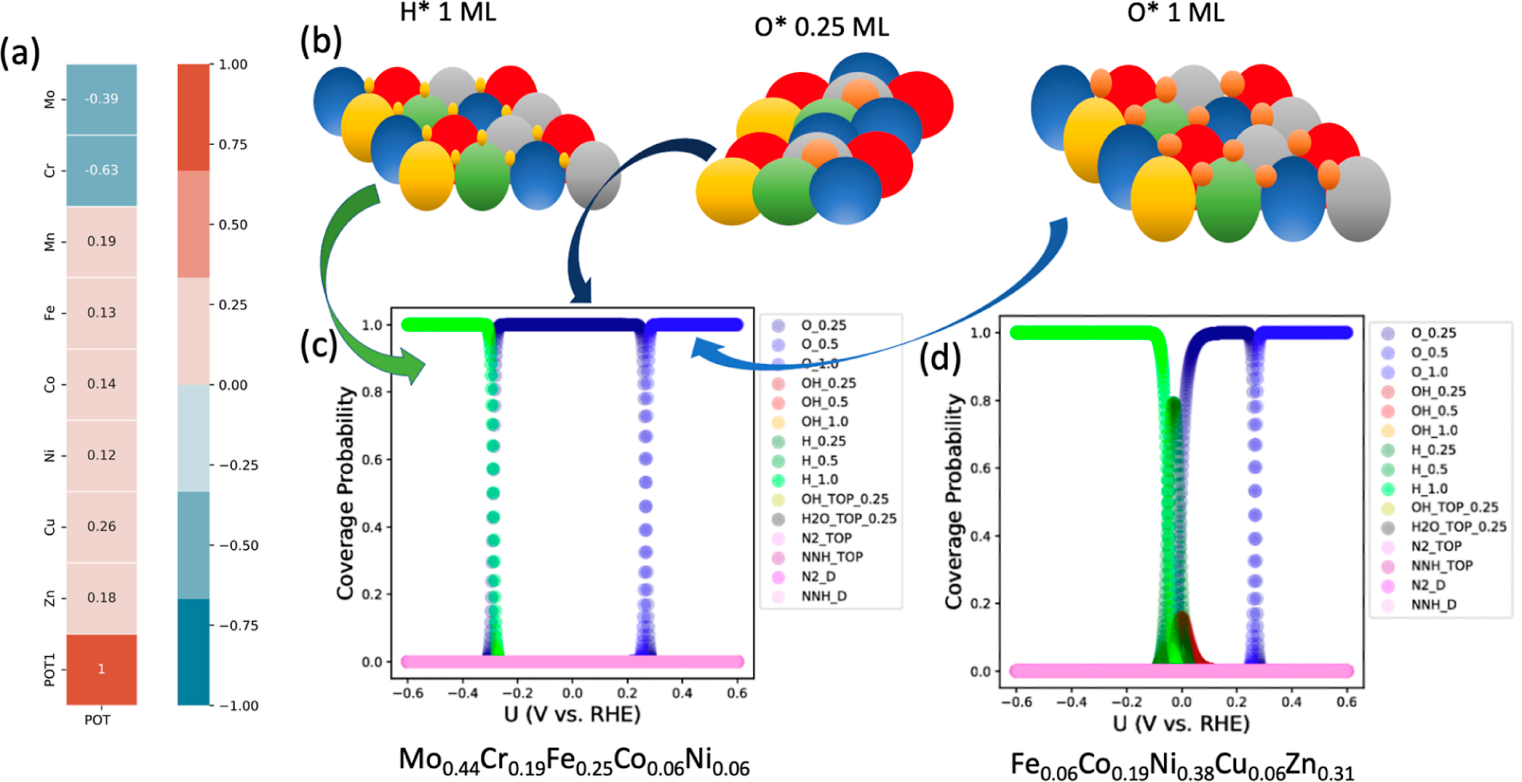
Linear correlation matrix between the element’s concentration and the potential where the surface coverage changes from hydrogenated to oxidized (point with higher probabilities to find N_2_ coverages) (a). Schematic picture of the surface coverages (b). Computed surface coverage probabilities of the materials Mo_0.44_Cr_0.19_Fe_0.25_Co_0.06_Ni_0.06_ (c) and Fe_0.06_Co_0.19_Ni_0.38_Cu_0.06_Zn_0.31_ (d). In the legend of (c,d), N2_D and NNH_D refer to the N_2_ horizontal and NNH horizontal.

Figure 4a presents a correlation (Pearson correlation coefficient) between the quinary HEA elemental concentrations investigated here and the potential versus RHE where the N_2_ coverages are maximized. The observed highest value of *R* is 0.26, represented by light orange for Cu, while the lowest value is −0.63, represented by dark orange for Cr. This indicates that a high concentration of later transition metals like Cu in the HEA composition pushes this potential to more positive values and vice versa. As expected, later transition metals with fully populated 3d states deliver lower adsorption energies for adsorbates, e.g., H* and O*. While this is true, the variation of adsorption energies with the elemental concentration of HEA for H* and O* might differ, and this results in distinct applied potentials where the surface coverage would be more likely populated by H* or O*. The lowest potential observed among the 9668 HEAs is −0.28 versus RHE for Mo_0.44_Cr_0.19_Fe_0.25_Co_0.06_Ni_0.06_, a HEA containing a great amount of earlier transition metals. On the other hand, the highest found value is −0.01 V versus RHE for Fe_0.06_Co_0.19_Ni_0.38_Cu_0.06_Zn_0.31_, a HEA containing a higher concentration of later transition metals.

The coverages of the limit cases, Mo_0.44_Cr_0.19_Fe_0.25_Co_0.06_Ni_0.06_ and Fe_0.06_Co_0.19_Ni_0.38_Cu_0.06_Zn_0.31_, are displayed in Figure 4c,d. For the former, from −0.6 V versus RHE until −0.28 V versus RHE, the probability of finding full H* surface coverage is 1.0 (probabilities vary from 0 to 1 as previously defined, and full coverage means 1 ML here). For the latter, the surface is fully covered with H* from −0.6 until −0.01 V versus RHE. Thus, all hollow sites are likely occupied by H* (Figure 4b) on the related potential region. Indeed, this is expected since the first step of HER—the Volmer step * + H_(aq)_^+^ + e^–^ → H*—is an electrochemical process; hence, negative potentials must increase the probability of surface hydrogenation in pro of the other considered states. From −0.28 V versus RHE until 0.25 V versus RHE, the surface is partially covered with 0.25 ML O* on the hollow positions for Mo_0.44_Cr_0.19_Fe_0.25_Co_0.06_Ni_0.06_. In the case of Fe_0.06_Co_0.19_Ni_0.38_Cu_0.06_Zn_0.31_, this situation changes, and other states like H* 0.25 ML coverage and OH* 0.25 ML coverage showed nonzero probabilities for potentials close to −0.01 V versus RHE. However, just after 0.0 V versus RHE, the 0.25 ML O* is the main coverage. For both cases, full oxidation of the surface is obtained for potentials higher than 0.25 V versus RHE. This is also expected since more positive potentials increase the Gibbs free energy of O* and OH* species, therefore, oxidizing the surface.

We have plotted the probabilities of coverage separately for all the considered coverage states in the simulation for Mo_0.44_Cr_0.19_Fe_0.25_Co_0.06_Ni_0.06_. Interestingly, the probabilities of finding a N_2_ coverage in the horizontal or vertical position exist for a very specific range of potentials, with the maximum value being at −0.28 V versus RHE (Figure S3). At this potential, a transition from a more likely hydrogenated surface to a more likely oxidized surface state exists and, hence, permits a nonzero probability of finding N_2_ on the catalytic surface.

**Figure 5:**
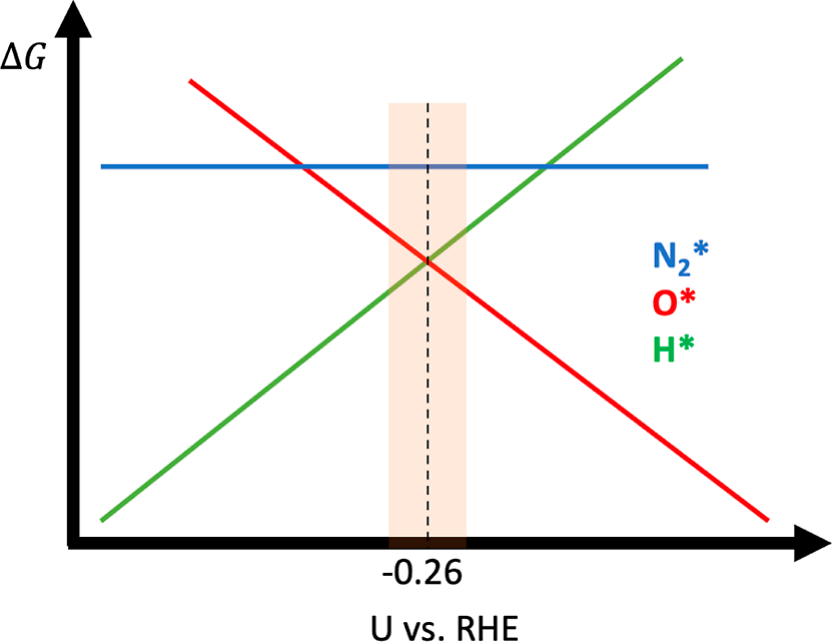
Schematic figure of the Gibbs free-energy variation in the computational hydrogen electrode approach (meaning Δ*G* varies with −*neU*_RHE_).

A schematic figure of the Gibbs free-energy variation in the computational hydrogen electrode approach^23^ (meaning Δ*G* varies with −*neU*_RHE_) brings up insights into what is happening (Figure 5). First, N_2_ adsorption strength does not depend on the applied potential. For the cases H* and O*, a linear relation exists but with opposite signs of angular coefficients. Therefore, there will exist a point where Δ*G*(H*) and Δ*G*(O*) are the same, and, at this point, the difference between Δ*G*(N_2_*) and Δ*G*(H*) or Δ*G*(O*) reaches a minimum. Thus, a higher probability of finding N_2_ adsorbed at this point is expected. For all the other potentials, the probabilities rapidly go to zero. Though we focused this part of the discussion on a specific HEA, Mo_0.44_Cr_0.19_Fe_0.25_Co_0.06_Ni_0.06_, this observation is general and appears similar to all 9668 cases observed here. This confirms the strong correlation; therefore, a coverage transition from a hydrogenated to an oxidized surface enables the N_2_ approach on the catalytic surface. This also explains why the more likely potentials for N_2_ adsorption vary with the elemental concentration, as previously discussed.

### 3.2 Catalytic Activities

CAs for the NNR reaction were computed for 9668 quinary HEAs formed with Mo–Mn–Fe–Co–Ni–Cu–Zn and divided into two cases: (1) the case where the NRR pathway is enzymatic and (2) the case where the NRR pathway is distal/alternating (Figure 2). For the first case, N_2_ adsorption occurs in the vertical position at the top site of the catalytic surface. This is followed by N_2_ hydrogenation also in the vertical position and still on the top site. For the latter, N_2_ adsorbs in the horizontal position at the “bridge site” of the catalytic surface and follows a H^+^ attack on the same site. Indeed, simultaneously, elemental optimization for both pathways is impossible since optimizing one pathway automatically leads to nonactive pathways in the other option. Hence, this is the main reason to divide the catalyst optimization into two parts (discussed further in the text).

Identifying the relationships between the HEAs’ intrinsic properties and their catalytic activity is also a way to simplify the search for highly active HEAs for NRR. We have recently shown that, for gas-phase cells, there exists a relationship between the delivered activity versus HAE’s VEC and ELE. Though this is true for that specific case, here, we introduce the effects of coverage due to the aqueous environment that can modify such relationships. HEA’s averaged properties like ELE, VEC, and WF are investigated and correlated to identify properties delimiting activities toward NRR.

#### 3.2.1 Enzymatic Pathway

A volcano-shaped relationship is found for CAs versus ELE and CAs versus VEC (Figure S4 and S5), while, for the plot of CAs versus WF, the volcano shape is partially broken (Figure S6). This means that, for VEC and ELE, there are optimal values delivering higher CAs (in this case, around 9.0 and 1.75 for VEC and ELE, respectively), while for WF, lower values lead to higher CAs. This is explained by checking separately the correlations between WF and the probabilities composing the CAs. In fact, the WF relationship with the N_2_ adsorption probabilities showed a point of maximum (volcano-shaped relation), while for the probability of H^+^ attacking the N_2_*-adsorbed molecule, a more linear trend is found (Figure S6). For the thermodynamical steps, no clear trend with WF is obtained (Figure S6). Therefore, WF only acts on the probabilities with a competitive character (steps 1 and 3 composing CAs in section Catalytic Activity), and the more linear relation comes from the H^+^-attacking probability.

For the case of VEC, a clearer relationship with the probabilities associated with the thermodynamical steps is obtained, while the competitive N_2_ adsorption and H^+^ attacks display a less clear trend (Figure S5). The point of maximum found on the CAs versus the VEC plot is where the probability of the two considered thermodynamical steps (step 2 in section Catalytic Activity) are balanced (a picture of the Sabatier principle) and, hence, filtered by the less correlated relation with the H^+^ attack. Though the H^+^ attack displays a less clear correlation, it still plays a role in constructing the final correlation of the CAs versus VEC (Figure S5). These trends obtained from VEC for the thermodynamical steps–meaning: the obtained probabilities of finding sites with exothermic reactions for the reactions N_2_* + H^+^ + e^–^ → NNH and NH* + H^+^ + e^–^ → NHH*—display that, for the earlier case, higher VEC leads to high probability, while the opposite for the later. This is associated with the electronic structures of the materials since the VEC is associated with the d-band filling, and this is known to correlate with the bond strength of intermediates on transition-metal surfaces.^37^ Since the first reaction is an activation process, stronger bonds are anticipated to deliver higher probabilities due to their exothermicity; hence, lower VEC delivers higher probabilities. For the later reaction, a desorption case, the opposite is true. Therefore, VEC is related to the bond strength of such intermediates, and this can be explained via the electronic structure of the transition metals and their d-band center positions.

**Figure 6:**
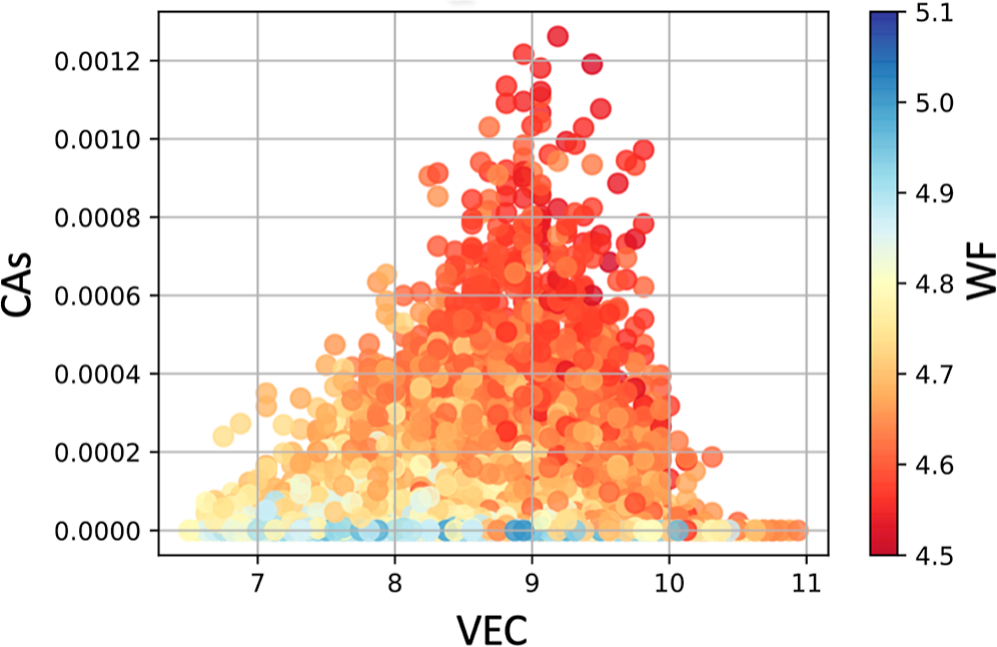
Relationships between CAs and VEC where color map displays the relationship with their (WF).

ELE has a similar relationship with probabilities to the case of WF but with less clear trends (Figure S4). Figure 6 shows the CAs versus VEC and, in the color map, the WF of each HEA. One can see that the HEA with the highest CA has a VEC of 9.18 and a WF of 4.5. For other HEAs presenting VEC of 9.18, but still with WF higher than 4.5, the resulting value of CAs is lower. Therefore, VEC and WF act as necessary conditions to increase the likelihood of activity toward NRR when NRR follows an enzymatic path in aqueous environment.

**Figure 7:**
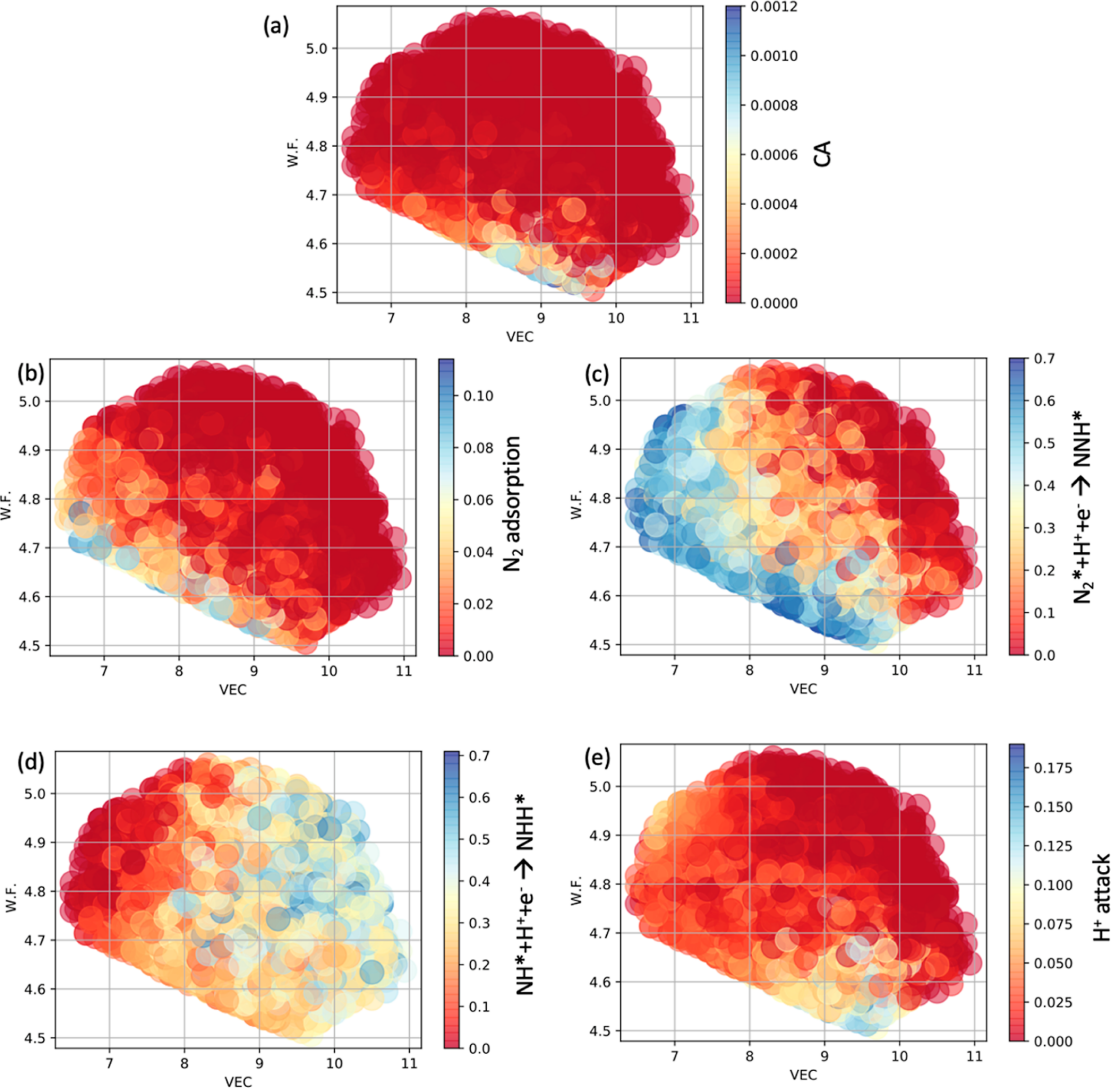
Relationships between WF and VEC for all the 9668 HEAs considered here. Color maps display the relationship with their (a) CAs, (b) probabilities of finding sites where N_2_ adsorbs exothermically and stronger than the competing species OH*, O*, and H*. (c) Probabilities of finding sites where N_2_* + H^+^ + e^–^ → NNH* is exothermic, (d) probabilities of finding sites where NH* + H^+^ + e– → NNH* is exothermic, and (e) probabilities of finding sites where H^+^ attack prefers to form NNH* than other competitive possibilities like OH* and goes to the surface forming H*.

The relationships between the probabilities and the intrinsic properties of the HEAs for the enzymatic pathway reveal that there are two main bottlenecks to achieve higher CAs that are the competitive N_2_ adsorption and the competitive H^+^ attack (Figure 7). The adsorption of N_2_ competes here with the adsorption of O* in the hollow position, OH* in the hollow position, and also H* in the hollow position. It becomes, therefore, an unlikely process to find specific sites where N_2_* adsorbs stronger than these counterparts. This is reflected in Figure 7b in which most of the HEAs displayed low probabilities of N_2_ adsorption (red color), and the cases with higher probabilities (light blue) have low values of WF.

The H^+^ attack also comes as a bottleneck to achieve high CAs. In general, H^+^ tends to go to the surface creating an H* coverage with minor probability of attacking a N_2_*-adsorbed molecule. This is one of the reasons why H_2_ is one of the main side products—this creates a competitive relationship between the hydrogen evolution reaction (HER) and NRR and moreover surface poisoning. HEAs with large amounts of later transition metals like Zn tend to present less affinity toward H* adsorption,^38,39^ therefore producing a higher probability of finding sites where H^+^ prefers to attack N_2_* than goes to the catalytic surface. Clearly, cases presenting higher H^+^ attack probabilities (light blue in Figure 7e) display, in general, WF lower than 4.6, and the element here presenting the lowest WF is Zn. It turns out that most HEAs with high CAs have a high content of Zn (see database file). In this regard, the NRR mechanism would follow similar to the case of single-atom catalysts where the active sites for NRR are surrounded by structures with low affinity toward H^+^.^40,41^ Thus, higher CAs are found for HEAs with lower WF.

Though important, the noncompetitive part of CAs (the thermodynamical steps N_2_* + H^+^ + e^–^ → NNH and NH* + H^+^ + e^–^ → NNH*) displayed much more cases with high probabilities (more cases presenting light blue, Figure 7c,d) than the competitive probabilities composed by N_2_ adsorption and H^+^ attack (more cases in red). This indicates that the competitive reactions are the bottleneck to finding HEAs with high CAs.

#### 3.2.2 Distal/Alternating Pathway

A volcano-shaped relationship is also found for CAs versus ELE and CAs versus VEC (Figures S7 and S8) for the distal/alternating pathways. However, different from the previous case, color mapping with the WF of each HEA does not lead to clear trends. Hence, these cannot uniquely describe the CAs, and selecting optimal HEAs based on these properties is not possible. Araujo et al.^18^ have recently shown that VEC and ELE can describe the catalytic activities toward NRR in a nonaqueous environment. However, when considering an aqueous environment, the competitive adsorption between OH* and N_2_* on the top sites of the catalytic surfaces needs to be considered. This breaks the correlations between ELE and VEC with the N_2_* adsorption and, thus, produces lower activities based on the probabilistic approach. For instance, in a nonaqueous environment, Mo captures N_2_ molecules due to their high interaction (related to Mo’s d-band center positioning).^37^ However, in a water environment, OH^–^ tends to also bond strongly on Mo sites, hence, preventing these from fixing the N_2_ molecules.

**Figure 8:**
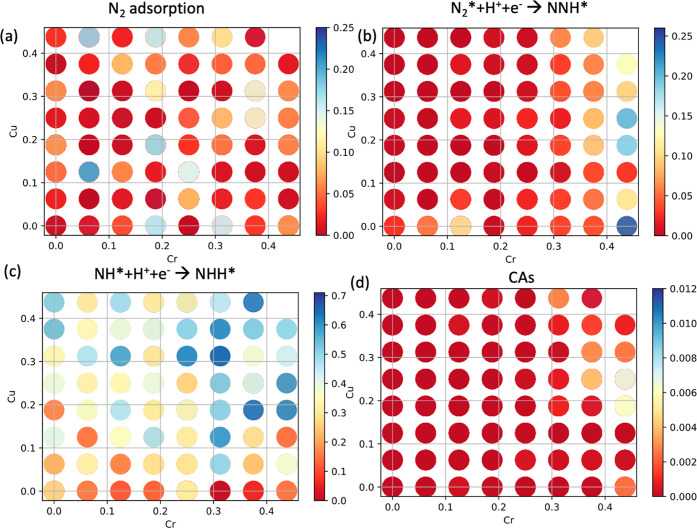
Probabilities of finding sites where N_2_ adsorbs exothermically and stronger than the competing species OH*, O*, and H* (a). Probabilities of finding sites where N_2_* + H^+^ + e^–^ → NNH* is exothermic (b). Probabilities of finding sites where NH* + H^+^ + e– → NNH* is exothermic (c). ,(d) Relationships between Cr and Cu elemental concentrations with CAs for all the 9668 HEAs considered here.

Back to the elemental concentration, CAs showed a clear dependence with the Cr and Cu concentrations on the referent HEA, where only cases with high Cr and Cu concentrations delivered high CAs (Figure S9). To sketch this, we have plotted the Cr concentration versus Cu concentration of all 9668 HEA investigated here (Figure 8d). Since the range of concentrations does not vary continuously, the result is a graphic with circles, where each circle represents a combination between Cr and Cu. Further, we color-mapped the CAs of each HEA, considering the multidimensionality of the data. The colors in Figure 8 represent the averaged CAs of HEAs with specific concentrations of Cr and Cu. Clearly, high CAs (yellow to light blue color) are for cases with the maximum allowed Cr concentration and a Cu concentration of 0.25. Hence, Cr and Cu concentrations emerge as a necessary condition to active high probabilities toward the NRR activity.

The relationships between the probabilities used to calculate CAs for the distal/alternating pathways reveal that the activation of the vertically adsorbed N_2_ molecule to form NNH* is the main reason for such trends. Higher concentration of Cr leads to higher probabilities of finding sites, where N_2_* + H^+^ + e^–^ → NNH* is exothermic (Figure 8b). On the other hand, the higher concentration of copper leads to higher probabilities of finding sites, where the reaction NH* + H^+^ + e^–^ → NNH* is exothermic. Hence, the balance between them delivers the final need for Cr and Cu concentrations for the catalytic activity for NNR in the distal/alternating pathway, and moreover, the bottleneck toward high activities for this pathway is the N_2_ vertical activation to form NNH* vertical. The other elements did not show such behavior (Figure S9).

### 3.3 Selected Cases

Though different pathways for the NRR lead to different HEAs as optimum candidates, the existence of both pathways in the same HEA may also be possible. To seek a HEA providing the highest performance toward NRR possible in both pathways, the CAs found for the enzymatic and distal/alternating pathways were added. This task was performed by normalizing the CAs of each pathway for values between 0 and 1 (avoiding data with orders of difference due to distinct treatment) and further adding these for each of the HEAs. The results showed that, in the newer rank, the same HEAs emerged as the top two as in the preview cases. This means that it is not possible to find HEA elemental concentrations that can optimize both pathways at the same time. Therefore, two cases are selected here for further analysis: the case showing the highest CA for the enzymatic NRR pathway and the case showing the highest CA for the distal/alternating pathway.

For the enzymatic pathway, the best HEA is formed by Mo_0.125_Cr_0.125_Mn_0.062_Fe_0.25_Zn_0.437_. Similar to the previously investigated coverage cases (section Coverages), this HEA surface is fully covered by H* for potentials lower than −0.25 versus RHE. From −0.25 until 0.25 versus RHE, the surface is covered by 0.25 ML O* occupying the hollow positions, while the left atoms (not in the vicinity of O*) are covered with OH* on the top. The surface is fully covered for more positive potentials versus RHE (Figure S10). The probability of finding N_2_ adsorbed on the surface of this HEA is the highest at −0.25 versus RHE—the potential where the surface suffers a coverage transition from hydrogenated to oxidized (Figure S11).

**Figure 9:**
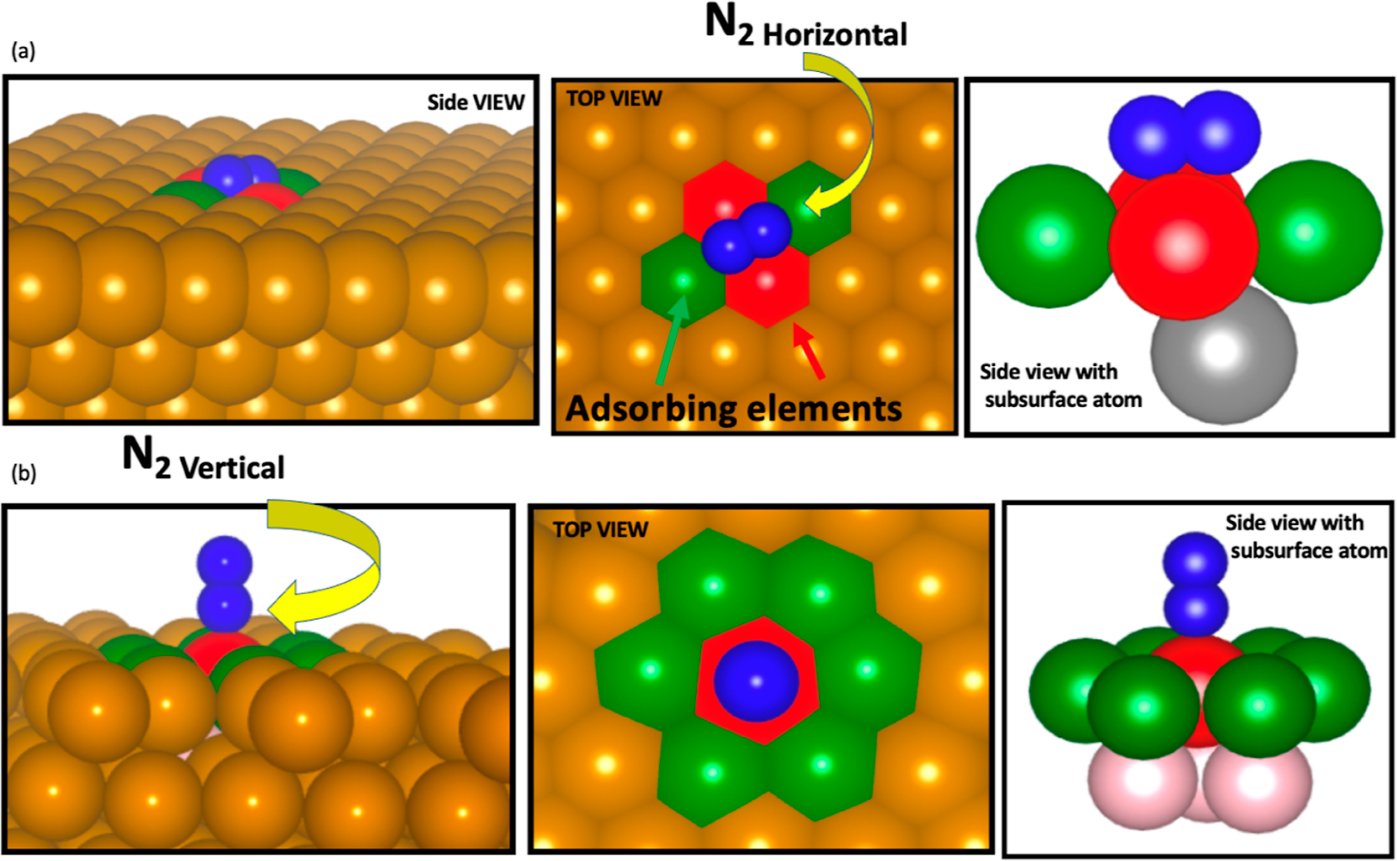
Schematic figure of the catalytic sites where N_2_ adsorbs horizontally (a). Schematic figure of the catalytic sites where N_2_ adsorbs vertically (b).

Based on the probabilistic approach, N_2_ adsorption on the catalytic surface is one of the bottlenecks to achieving high NRR efficiency for Mo_0.125_Cr_0.125_Mn_0.062_Fe_0.25_Zn_0.437_. As already mentioned, this is due to the higher surface species interaction with intermediates like O*, OH*, and H* that competes with the dinitrogen adsorption, hence, poisoning the catalytic surface or leading to HER instead of NRR at such potential. For the 2000 microstates calculated for the specific HEA Mo_0.125_Cr_0.125_Mn_0.062_Fe_0.25_Zn_0.437_, only 112 cases displayed a preference to adsorb N_2_ instead of the competing counterparts. Figure 9a represents the catalytic site where N_2_ adsorbs horizontally on an FCC (111) surface where elements among Mo–Cr–Mn–Fe–Co–Ni–Cu–Zn can populate the green sites, red sites, and the gray subsurface site of the HEA lattice. From the 112 cases where N_2_ preferentially gets adsorbed, 57% of green sites are populated with Fe, 16% with Cr and Mn, while 10% with Zn. Other elements showed a lower percentage. Red sites are 51% populated by Cr, 26% populated by Mo, and 16% by Fe. Other elements appear with minor probabilities. These results show that the catalytic sites competitively adsorbing N_2_ are mostly formed by Fe in the green sites, while the red sites are mostly formed by Cr, even though the elemental concentration of Zn in the referent HEA is the highest. The subsurface site on the HCP position also showed elemental preference with about 20% of Mo, Cr, and Mn and 35% of Zn. Interestingly, Mn concentration in this material is very low and still, for this case, 20% of the lattice sites are populated with Mn. This highlights the importance of the subsurface element in the HCP sites toward the preferential adsorption of N_2_ at the catalytic surface.

For the distal/alternating pathway, the best HEA is Mo_0.06_Cr_0.44_Co_0.125_Ni_0.06_Cu_0.31_. The surface coverage follows the same behavior as for the case of Mo_0.125_Cr_0.125_Mn_0.062_Fe_0.25_Zn_0.437_, where the surface is fully hydrogenated for potentials before −0.25 V versus RHE, partially oxidized for potentials that are between −0.25 and 0.25 V versus RHE and fully oxidized for more positive potentials (Figure S12).

Based on the probabilistic approach, N_2_ adsorption in the vertical position has a higher probability of occurrence than that presented by horizontal N_2_ adsorption since the competition, in this case, is only with OH* on the top. However,, for Mo_0.06_Cr_0.44_Co_0.125_Ni_0.06_Cu_0.31_, it is the bottleneck to achieving higher CAs. Figure 9b represents the catalytic site where N_2_ adsorbs vertically on an FCC(111) surface where elements among Mo–Cr–Mn–Fe–Co–Ni–Cu–Zn can populate the green sites, red sites, and the pink subsurface site of the HEA lattice. From the 2000 microstates calculated for the Mo_0.06_Cr_0.44_Co_0.125_Ni_0.06_Cu_0.31_ HEA, 264 cases showed N_2_ adsorption on the top stronger than OH* adsorption. From these cases, 30% of the N_2_-bonding element (red in Figure 9a) is populated with Mo, 29% with Cr, and 40% with Co. These are, therefore, the attractive centers for N_2_ fixation. Interestingly, Cr is the element with the highest concentration in this HEA; hence, Cr might be expected to populate the red sites among the 264 cases within the same proportion as in the HEA. However, the majority of the red sites from the 264 cases are formed by Co-element with a lower general concentration. The green sites are populated such that 50% of the 264 cases are by Cu, 20% by Co, and 15% by Ni and Cr. Also here, Cu has about 30% of the elemental concentration of this HEA, but it populates 50% of the green sites. Finally, the subsurface sites (pink in Figure 9b) of the 264 cases are mostly populated by Cr and Cu.

## 4 Discussion and Concluding Remarks

This work developed an efficient strategy to model and screen aqueous NRR-efficient five-element HEA catalysts formed by elements in the Mo–Cr–Mn–Fe–Co–Ni–Cu–Zn series. Our results show that, at the vast majority of the applied potentials, the catalytic surfaces are covered by oxide groups (O* and OH*) or are hydrogenated. The surface coverages, along with the N_2_ triple bonds and the lack of dipole moments of N_2_, lead to small probabilities of N_2_ fixation on the catalytic surfaces, leading to low activities toward NRR. However, there exists a specific potential where the surface coverage transforms from a hydrogenated state to a more oxidized state and is identified as the key potential that one should find for the given catalyst. At this specific point, the probability of N_2_ coverage increases. Our results suggest that, for NRR in the enzymatic pathway, selecting HEA’s averaged valence electron concentration and their averaged work function can increase the probability of catalytic activity. Moreover, the bottlenecks to find HEAs with high catalytic activities are the competitive relations and not the thermodynamical steps. This means: N_2_ adsorption versus the adsorption of species like O*, OH*, and H* together with the lower probabilities of H^+^ attacking the N_2_ adsorbed versus the probability of forming OH* or just going to the surface forming H* is between the determinants for the very low activities found experimentally. Different relationships were found for the case of distal/alternating pathway. There, the concentration of Cr and Cu emerged as the main driving parameters toward high activities. Moreover, the first hydrogenation process, the formation of NNH*, appears as the bottleneck (distal N_2_ molecules are less polarized than the enzymatic N_2_ molecules, therefore leading to higher thermodynamical steps to form NNH*). We pointed to the HEA, Mo_0.125_Cr_0.125_Mn_0.062_Fe_0.25_Zn_0.437_, as the best option for the enzymatic pathway, while Mo_0.06_Cr_0.44_Co_0.125_Ni_0.06_Cu_0.31_ as the best for the distal/alternating path.

Our results disclose meaningful relationships attributed to the materials’ properties that can be used to design active HEA catalysts for NRR in aqueous environment under competitive surface adsorption processes. Based on such results, one can build the hypothesis that adding elements with even lower WFs than the ones used here would further improve the selectivity and activity by enhancing the probabilities of H^+^ attack on the enzymatic N_2_-adsorbed molecules, for instance. The experimental values of the WFs were among the first descriptors for HER.^42^ Kani et al.^43^ also hypothesized that the most efficient catalyst for NRR would be the one with a lower hydrogen adsorption H*, providing lower H coverage. This concept is realized in the work of Hao et al.,^44^ which reported a high FE of 66% for NNR on a Bi catalyst. Bi is known to be a HER poisoner,^45^ and the outstanding performance of such catalysts is attributed to the lower affinity toward H*, the reasonable thermodynamical step to form NNH*, and also the presence of high concentrations of K^+^ cation preventing the high concentration of H^+^ close to the catalytic surface—that can deteriorate the N_2_ fixation due to competition. Another solution toward higher activity and selectivity toward NRR is to add a proton donor controllability in a nonaqueous solution, such as the recent study showing high FE using ethanol as a proton donor and sacrificing agent in a tetrahydrofuran solution.^46^ The use and consumption of a high-value chemical as a proton source to produce low-molecular-weight ammonia, however, do not form a sustainable solution. Another route, viable in any solvent, would be to introduce p group metals in the catalyst, like Bi, in the HEA.^44^ This could deplete H^+^ at the surface to slowdown HER, thus increasing the likelihood that H^+^ binds to N_2_, while the other HEA elements would still form N_2_-attracting centers. Moreover, adding a high molar concentration of KOH together with a portion of a solvent that increases the N_2_ solubility in water (N_2_ solubility in water is relatively low 1.3 × 10^–3^ mol/L^43^) might enhance the probability of achieving high FEs since the N_2_ molecule concentration would be higher close to the catalytic surface. Wang et al.^47^ have also reported an alternative approach that managed to deliver FEs of 71% and a rate of 9.5 × 10^–10^ mol s^–1^ cm^–2^ at −0.3 V versus RHE in an aqueous environment. This is based on electrolytes formed with a high salt concentration in water, of the order of 10 M. The high salt concentration controls the proton supply by increasing the number of water molecules in the cation hydration shell, in contrast with dilute cases where more free water is found. Moreover, they showed that N_2_ molecules tend to precipitate on the catalytic surfaces for the case of highly salt-concentrated electrolytes benefiting, hence, NRR processes.

In summary, a path toward a highly efficient electrocatalytic ammonia production would involve the benefits of HEA catalysts composed of a mixture of elements presenting very low WFs and N_2_-capturing elements together with an electrolyte optimization process. It is crucial to emphasize that this work has not uncovered a silver bullet solution for the electrochemical ammonia production. Instead, it has revealed the primary bottlenecks associated with achieving higher probability of activity for electrochemical ammonia production using high-entropy alloys (HEA). Notably, even in the most favorable scenario identified in this study, the probability of N_2_ coverage remains low. This underscores the fact that catalyst optimization alone is insufficient for achieving efficient NRR in an aqueous environment. This suggests that, for practical applications of such catalysts, a comprehensive approach from the experimental standpoint would require electrolyte/condition optimization to enhance the probabilities of N_2_ coverage. This explains the low activities of the order of 10^–11^ to 10^–12^ mol cm^–1^ s^–1^ found in difference experiments.^36^ Regarding the electrolyte, the application of highly concentrated salts in water emerged as a sustainable alternative as compared to the application of high-value chemicals as a proton source and still with the advantage of controlling the proton supply to prevent HER and increase the concentration of N_2_ in the catalyst–electrolyte interface. Increasing N_2_ pressure can also be a way to improve the water solubility, yielding N_2_ coverages.

It is important to note that several of the HEA concentrations suggested in this study significantly differ from the equimolar condition, which is optimal for maximizing the entropic effects stabilizing the HEA solid solution. The potential for HEA solid solution formation can be estimated using empirical data such as atomic sizes, formation enthalpy, and configurational entropy.^48^ When the terms  and  the HEA might form a solid solution. Here, δ is a parameter gauging the atomic size difference that depends on, *C*_*i*_, the atomic percentage of *i*th component, *r*_*i*_ atomic radius of *i*th component and *r*^ave^ the averaged atomic radius. Ω parameter depends on the concentration weighted averaged melting temperature, Tm, the configurational entropy Δ*S*_mix_ = −*R*∑_*i* = 1_^*N*^*C*_*i*_ ln *C*_*i*_ and mixing enthalpy Δ*H*_mix_ = ∑_*i*,*j*_^*N*^*C*_*i*_*C*_*j*_4*H*_*i*,*j*_ where *H*_*i*,*j*_ is the mixing enthalpy of binary alloys computed based on Miedema macroscopic model.^49^ While some of the investigated HEAs in this study deviate from equimolar elemental concentrations, reducing Δ*S*_mix_ and the likelihood of forming a solid solution, the primary objective of this study was to unravel the key relationships between NRR bottlenecks in an aqueous environment and the elemental concentration of HEAs.

## Supporting Information Available

ZPE and solvation energies of each intermediate used to correct the thermodynamical barriers, lattice constants, adsorption sites, DNN training models, surface coverage probabilities, relations between catalytic activities vs working function, and valence electron concentration and electronegativity (XLSX)
(XLSX)
(PDF)
