# Supplementary material for: Supervised AI and Deep Neural Networks to Evaluate High-Entropy Alloys as Reduction Catalysts in Aqueous Environments
Source: ACS Catal. 2024 Feb 22;14(6):3742–55. doi: 10.1021/acscatal.3c05017 (PMC10949192; doi:10.1021/acscatal.3c05017)
Supplement: Supplementary file 3 — cs3c05017_si_003.pdf [file cs3c05017_si_003.pdf]

Supplementary info for **“Supervised AI and Deep Neural Networks to Evaluate High Entropy Alloys as Reduction Catalysts in Aqueous Environments”**

Rafael B. Araujo[a,*] and Tomas Edvinsson[a,b*]

[a] *Department of Materials Science and Engineering, Solid State Physics, Uppsala University, Box 35, 75103 Uppsala, Sweden*

[b] *Energy Materials Laboratory, School of Natural and Environmental Science, Newcastle University,*
Newcastle Upon Tyne NE1 7RU, United Kingdom

[*] Corresponding authors: rafael.araujo@angstrom.uu.se, tomas.edvinsson@angstrom.uu.se

---

Table S1: ZPE and implicit solvation energies of each intermediate adsorbed on 10 randomly created microstructures and averaged. These values are further used to correct adsorption energies from the deep neural network.

Intermidiate	Delta ZPE	Delta_solv
NH_HPC	0.37	-0.07
NHH_bridge	0.89	-0.15
N_2_TOP	0.2	-0.1
N_2_H	0.17	-0.06
NNH_TOP	0.46	-0.17
NNH_H	0.48	-0.15
H_2O_TOP	0.62	-0.23
OH_TOP	0.33	-0.16
OH_HPC	0.37	-0.18
O_HCP	0.07	-0.06
H_HCP	0.17	-0.04

Table S2: Lattice constate values used to weight-average to assess the HEA lattice constants.

	Lattice Constant (Å)
Mo	3.99
Cr	3.61
Mn	3.50
Fe	3.65
Co	3.53
Ni	3.54
Cu	3.66
Zn	3.97

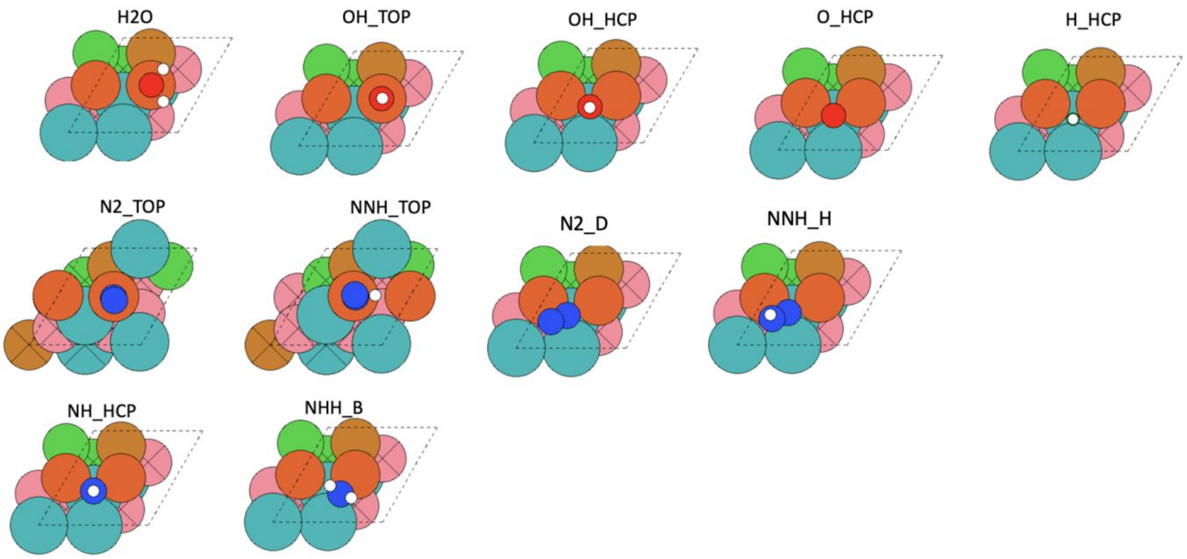

Figure S1: Adsorption configuration of the NRR used to build the neural networks. In this figure, the adsorbates are shown on one of the 1200 randomly created microstructures.

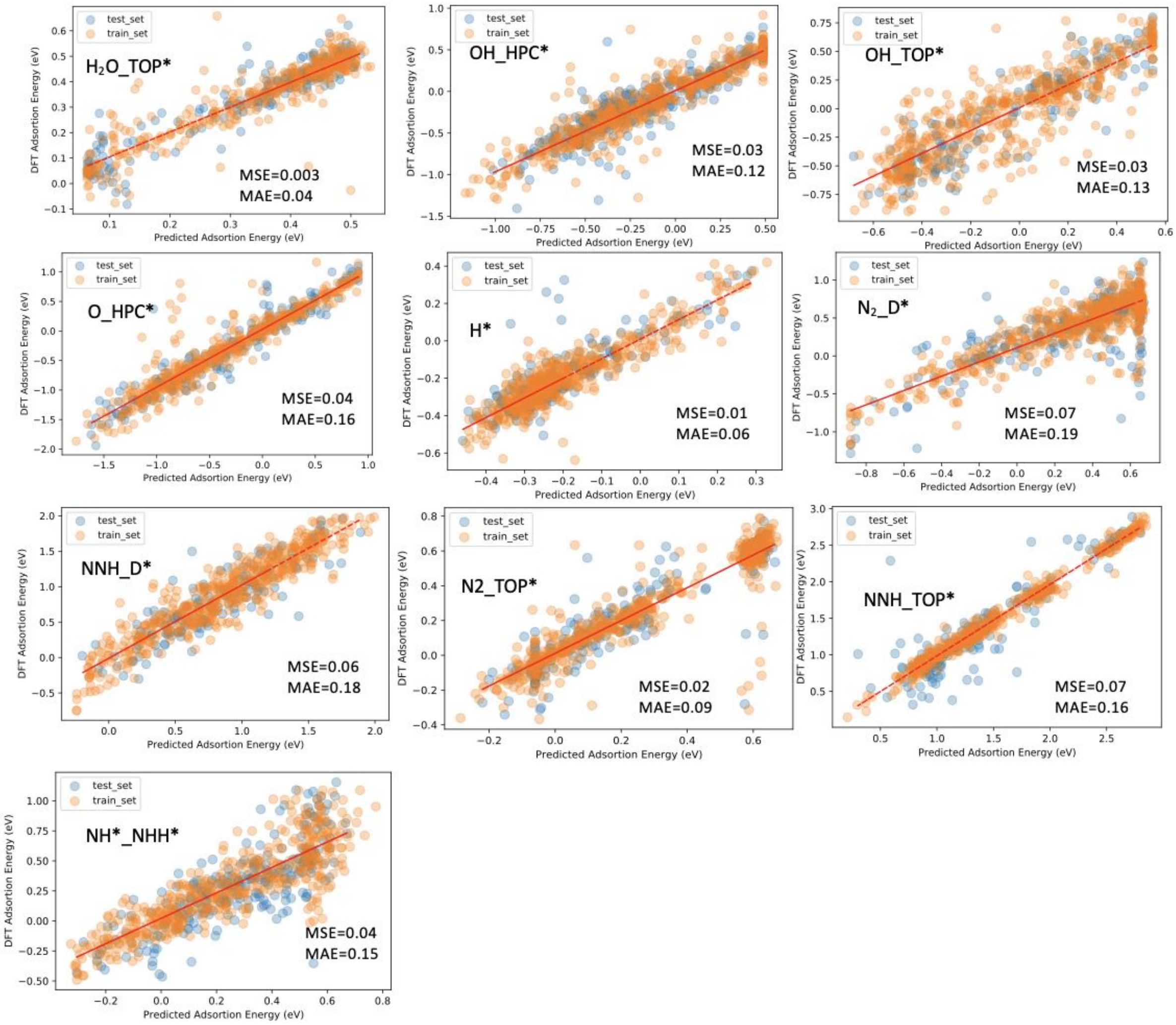

Figure S2: Comparison between the predicted adsorption energies with the DNN and the computed energies. Blue is the data used in the test set, while black dots are the data of the train set.

Model: "H_HPC"

Layer (type)	Output Shape	Param #
dense_19 (Dense)	(None, 420)	13860
dense_20 (Dense)	(None, 256)	107776
dense_21 (Dense)	(None, 128)	32896
dense_22 (Dense)	(None, 1)	129

Total params: 154,661
Trainable params: 154,661
Non-trainable params: 0

Model: "H2O_TOP"

Layer (type)	Output Shape	Param #
dense_45 (Dense)	(None, 320)	10560
dense_46 (Dense)	(None, 32)	10272
dense_47 (Dense)	(None, 32)	1056
dense_48 (Dense)	(None, 1)	33

Total params: 21,921
Trainable params: 21,921

Model: "N2_D"

Layer (type)	Output Shape	Param #
dense_186 (Dense)	(None, 256)	8448
dense_187 (Dense)	(None, 512)	131584
dense_188 (Dense)	(None, 128)	65664
dense_189 (Dense)	(None, 128)	16512

dense_190 (Dense)	(None, 320)	41280
dense_191 (Dense)	(None, 480)	154080
dense_192 (Dense)	(None, 64)	30784
dense_193 (Dense)	(None, 1)	65

Total params: 448,417
Trainable params: 448,417
Non-trainable params: 0

Model: "N2_TOP"

Layer (type)	Output Shape	Param #
dense_36 (Dense)	(None, 200)	6600
dense_37 (Dense)	(None, 128)	25728
dense_38 (Dense)	(None, 256)	33024
dense_39 (Dense)	(None, 128)	32896
dense_40 (Dense)	(None, 48)	6192
dense_41 (Dense)	(None, 1)	49

Total params: 104,489
Trainable params: 104,489
Non-trainable params: 0

Model: "NH_NHH"

Layer (type)	Output Shape	Param #
dense_235 (Dense)	(None, 380)	12540
dense_236 (Dense)	(None, 256)	97536
dense_237 (Dense)	(None, 128)	32896
dense_238 (Dense)	(None, 256)	33024
dense_239 (Dense)	(None, 1)	257

==

Total params: 176,253
Trainable params: 176,253
Non-trainable params: 0

Model: "NNH_D"

Layer (type)	*Output Shape*	*Param #*
dense_169 (Dense)	*(None, 320)*	*10560*
dense_170 (Dense)	*(None, 512)*	*164352*
dense_171 (Dense)	*(None, 256)*	*131328*
dense_172 (Dense)	*(None, 128)*	*32896*
dense_173 (Dense)	*(None, 1)*	*129*

Total params: 339,265
Trainable params: 339,265
Non-trainable params: 0

Model: "NNH_TOP"

Layer (type)	*Output Shape*	*Param #*
dense_39 (Dense)	*(None, 512)*	*16896*
dense_40 (Dense)	*(None, 300)*	*153900*
dense_41 (Dense)	*(None, 256)*	*77056*
dense_42 (Dense)	*(None, 1)*	*257*

Total params: 248,109
Trainable params: 248,109

Model: "O_HPC"

Layer (type)	*Output Shape*	*Param #*
dense_6 (Dense)	*(None, 256)*	*8448*
dense_7 (Dense)	*(None, 64)*	*16448*

```
dense_8 (Dense)           (None, 128)           8320
_________________________________________________________________
dense_9 (Dense)           (None, 256)           33024
_________________________________________________________________
dense_10 (Dense)          (None, 1)             257
=================================================================
Total params: 66,497
Trainable params: 66,497
Non-trainable params: 0
_________________________________________________________________

_________________________________________________________________
Model: "OH_HPC"
_________________________________________________________________
Layer (type)              Output Shape          Param #
=================================================================
dense_139 (Dense)         (None, 420)           13860
_________________________________________________________________
dense_140 (Dense)         (None, 256)           107776
_________________________________________________________________
dense_141 (Dense)         (None, 128)           32896
_________________________________________________________________
dense_142 (Dense)         (None, 256)           33024
_________________________________________________________________
dense_143 (Dense)         (None, 128)           32896
_________________________________________________________________
dense_144 (Dense)         (None, 256)           33024
_________________________________________________________________
dense_145 (Dense)         (None, 64)            16448
_________________________________________________________________
dense_146 (Dense)         (None, 1)             65
=================================================================
Total params: 269,989
Trainable params: 269,989
Non-trainable params: 0
_________________________________________________________________

Model: "OH_TOP"
_________________________________________________________________
Layer (type)              Output Shape          Param #
=================================================================
dense_383 (Dense)         (None, 512)           16896
_________________________________________________________________
dense_384 (Dense)         (None, 256)           131328
_________________________________________________________________
dense_385 (Dense)         (None, 256)           65792
_________________________________________________________________
dense_386 (Dense)         (None, 512)           131584
```

dense_387 (Dense)	*(None, 48)*	*24624*
dense_388 (Dense)	*(None, 1)*	*49*

Total params: 370,273
Trainable params: 370,273
Non-trainable params: 0

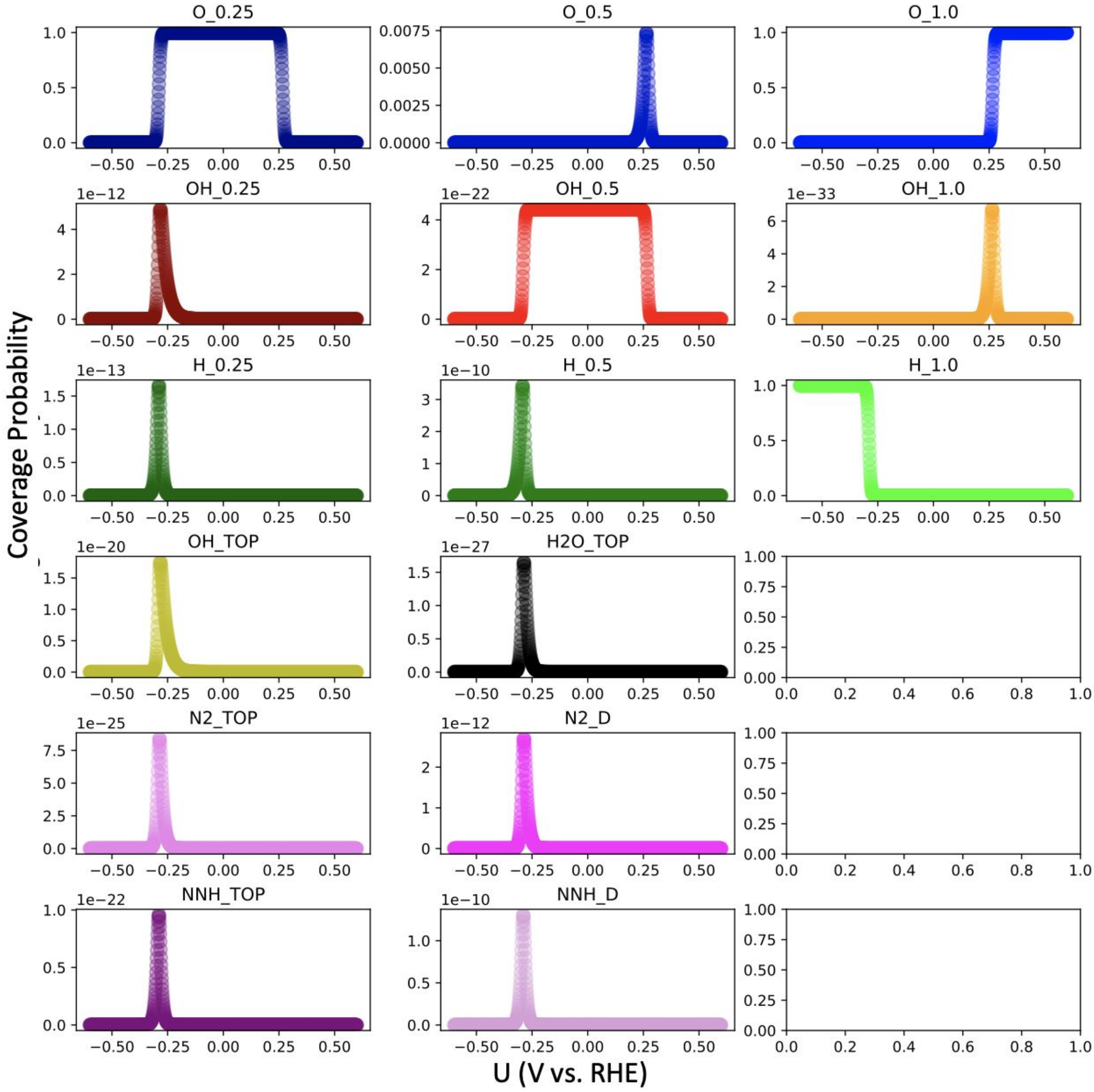

Figure S3: Surface coverages probabilities calculated as described in the section for $Mo_{0.44}Cr_{0.19}Fe_{0.25}Co_{0.06}Ni_{0.06}$.

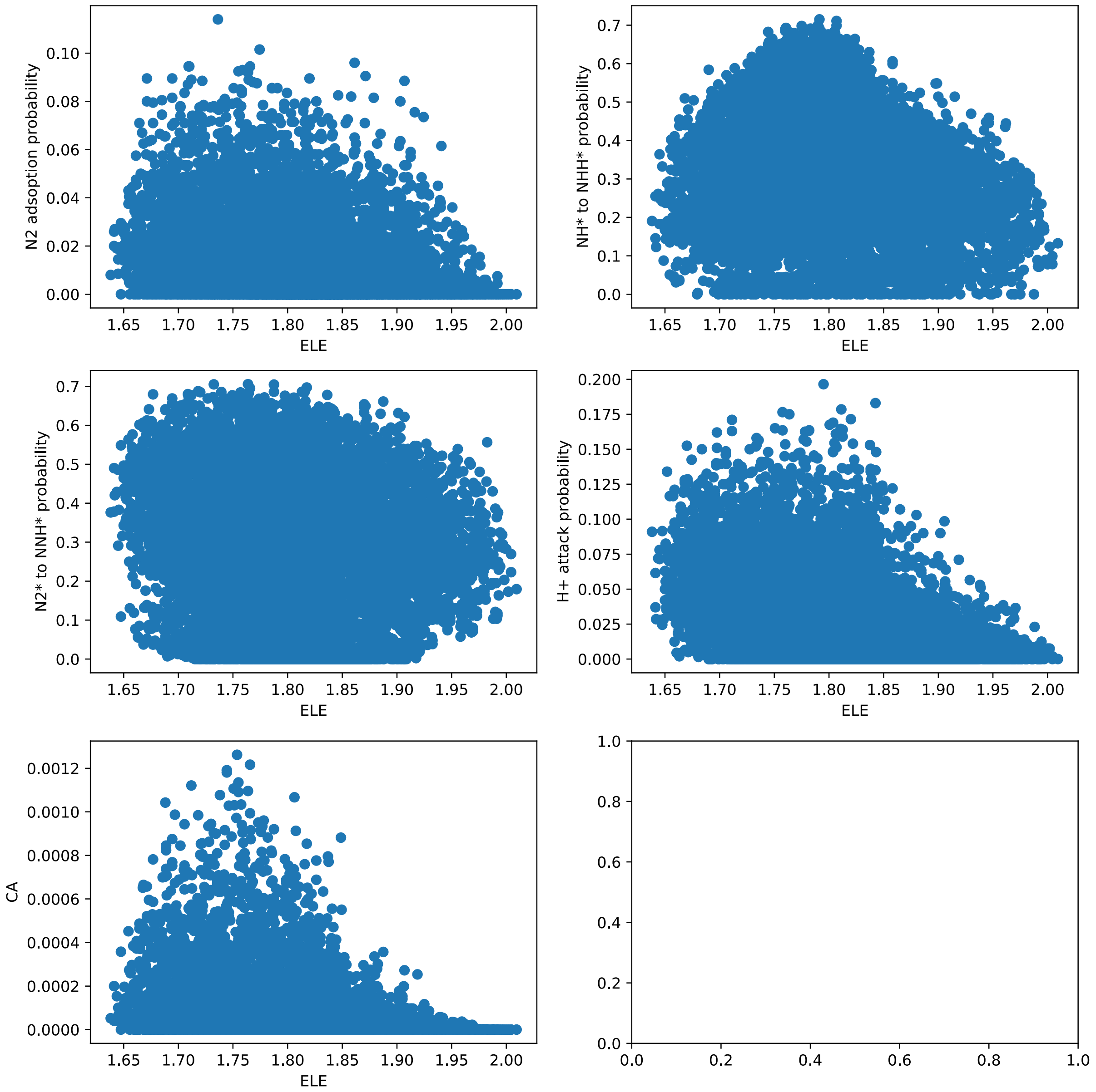

Figure S4: Relationships between the probabilities used to compute CAs and ELE of the HEA in the enzymatic pathways.

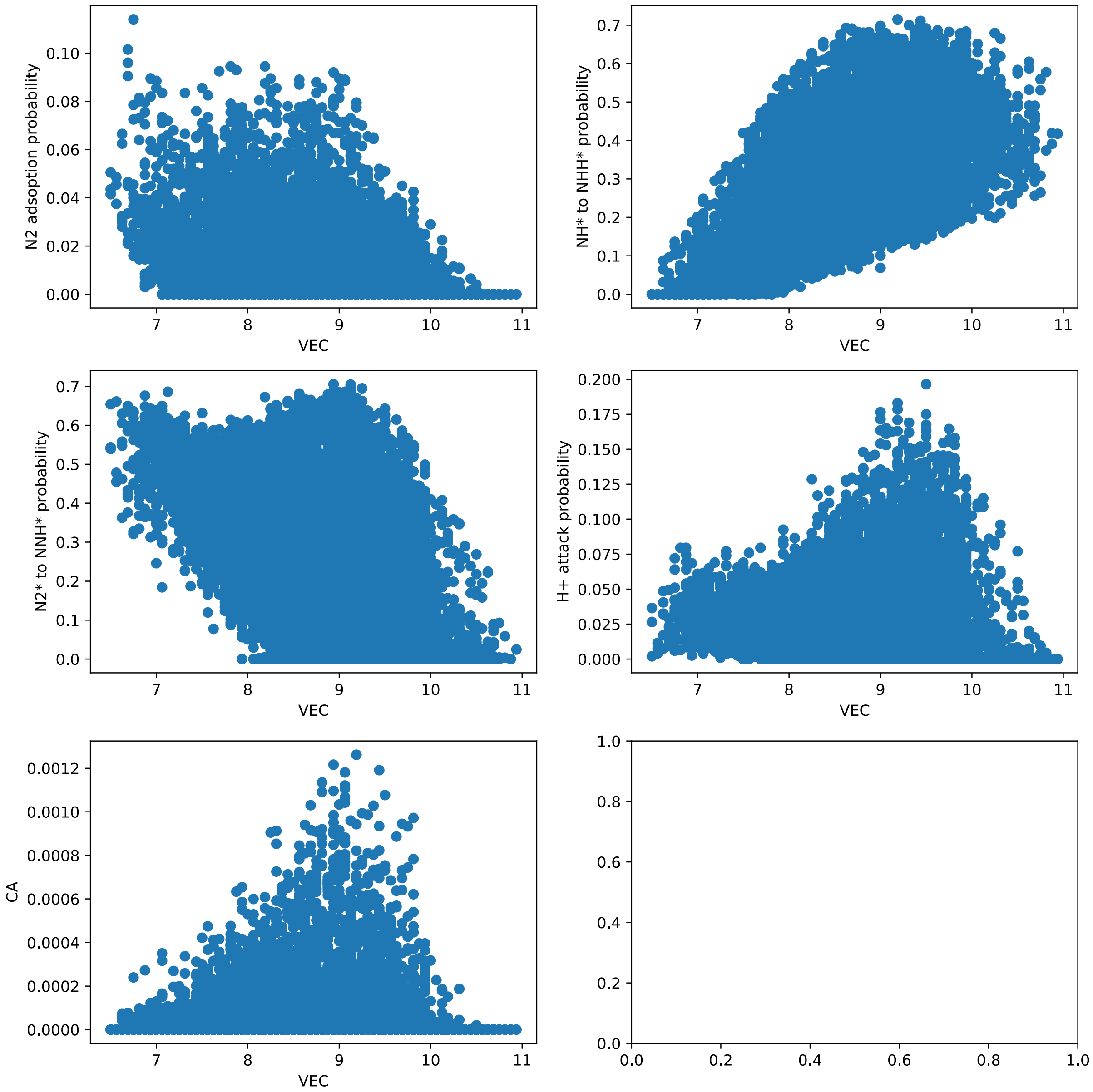

Figure S5: Relationships between the probabilities used to compute CAs and VEC of the HEA in the enzymatic pathways.

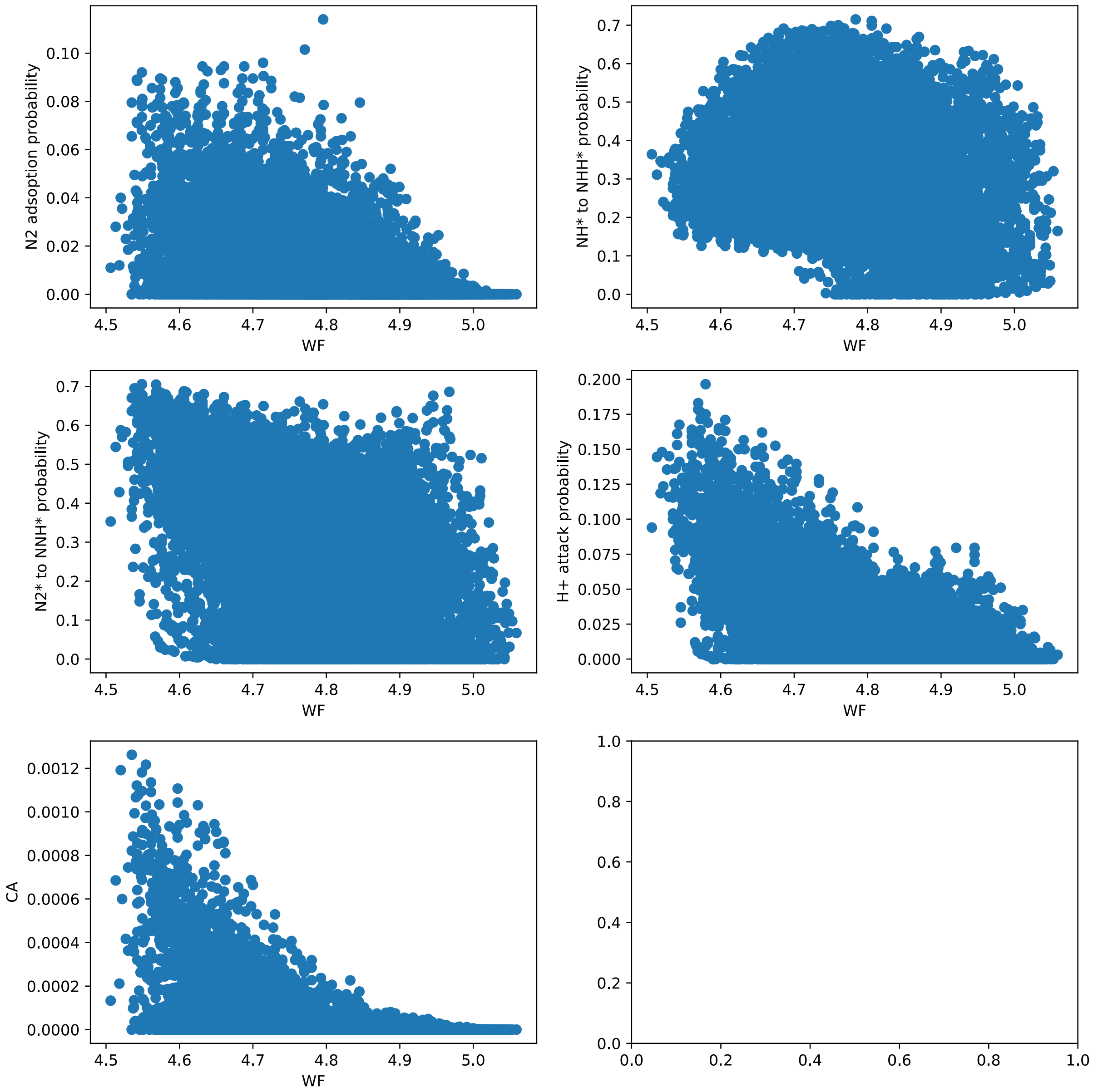

Figure S6: Relationships between the probabilities used to compute CAs and WF of the HEA in the enzymatic pathways.

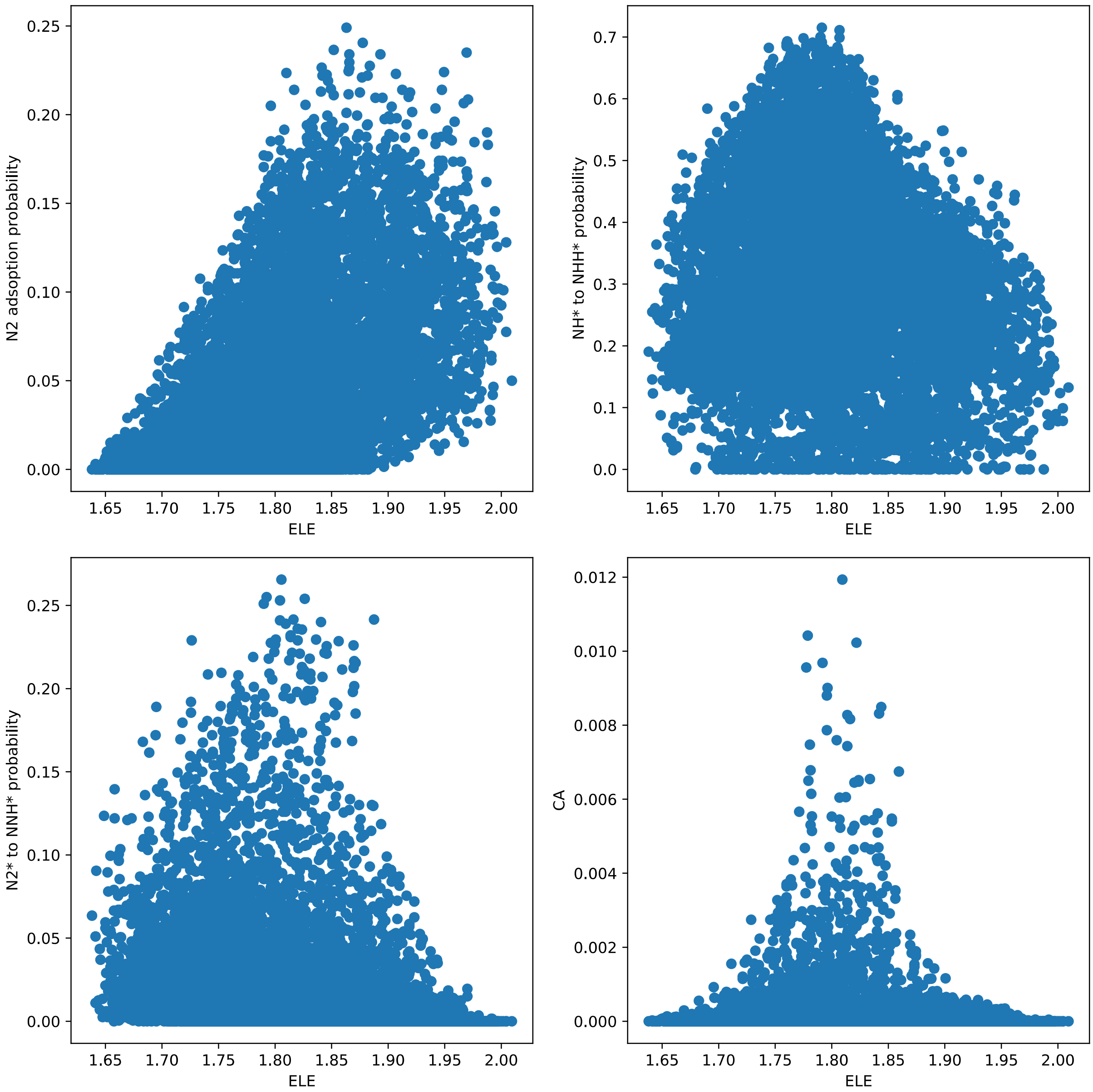

Figure S7: Relationships between the probabilities used to compute CAs and ELE of the HEA in the distal/Alternating pathways.

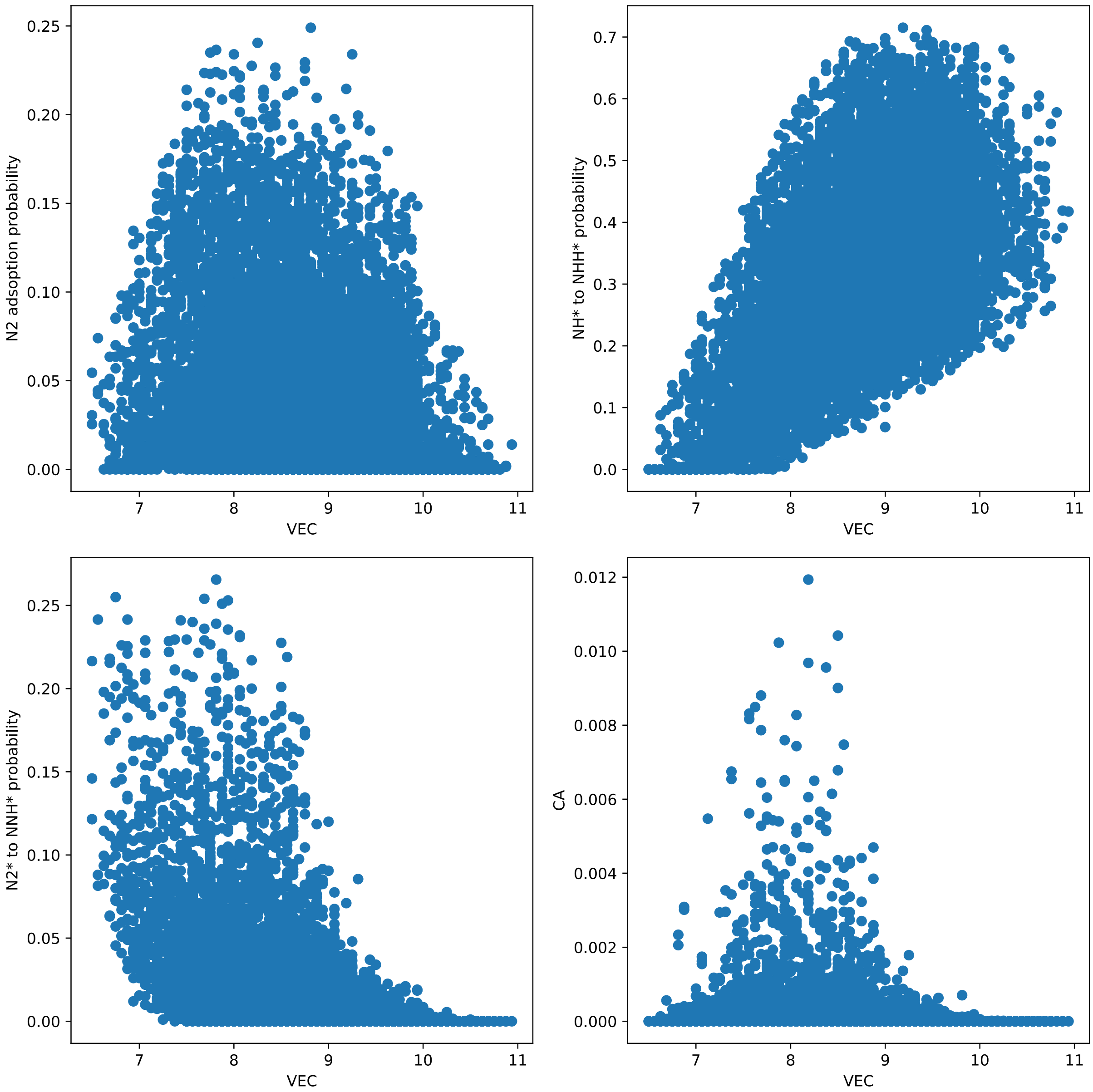

Figure S8: Relationships between the probabilities used to compute CAs and VEC of the HEA in the distal/Alternating pathways.

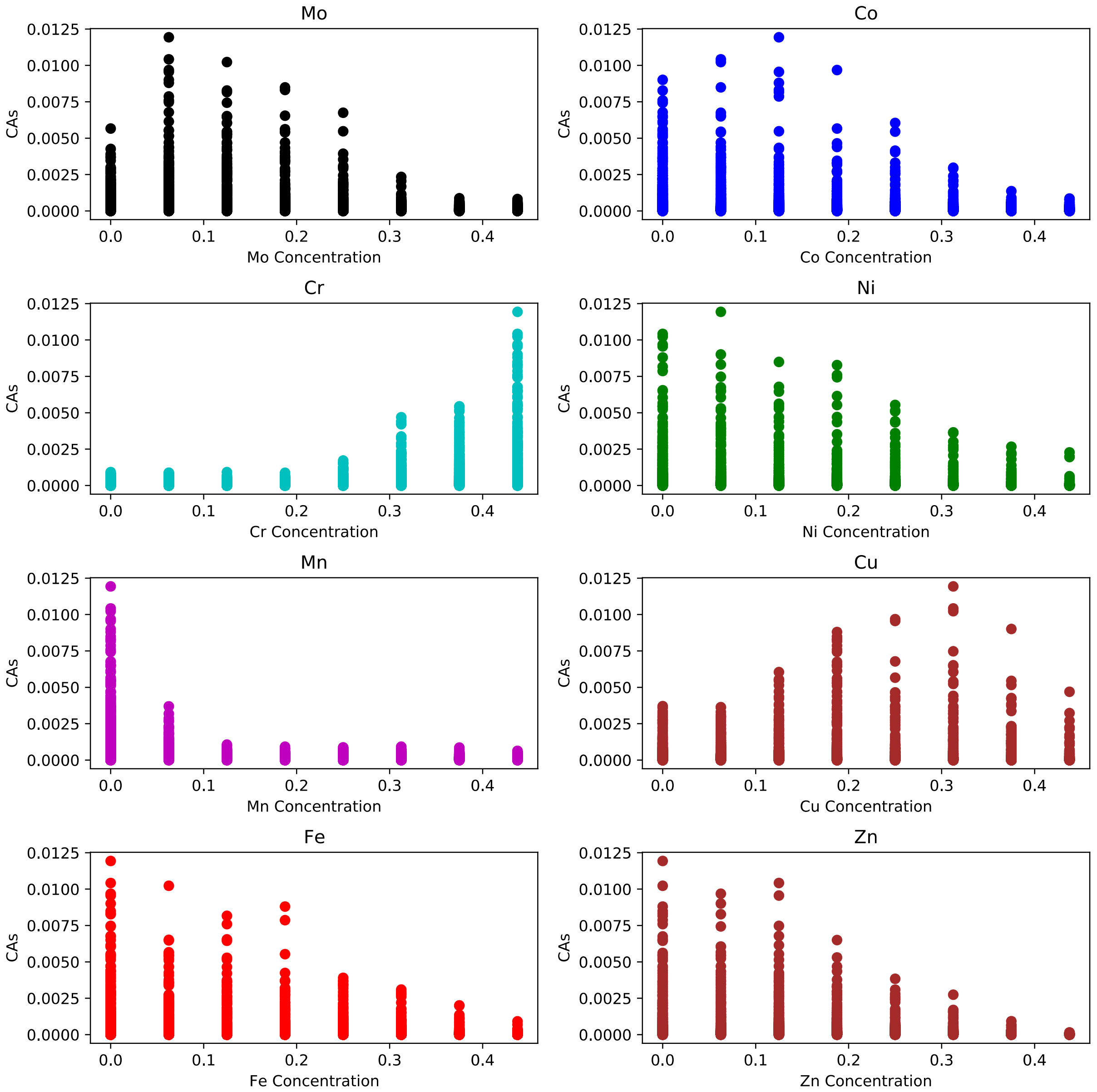

Figure S9: The relationships between the probabilities used to calculate CAs for the distal/alternating pathways and the elemental concentrations of the HEAs.

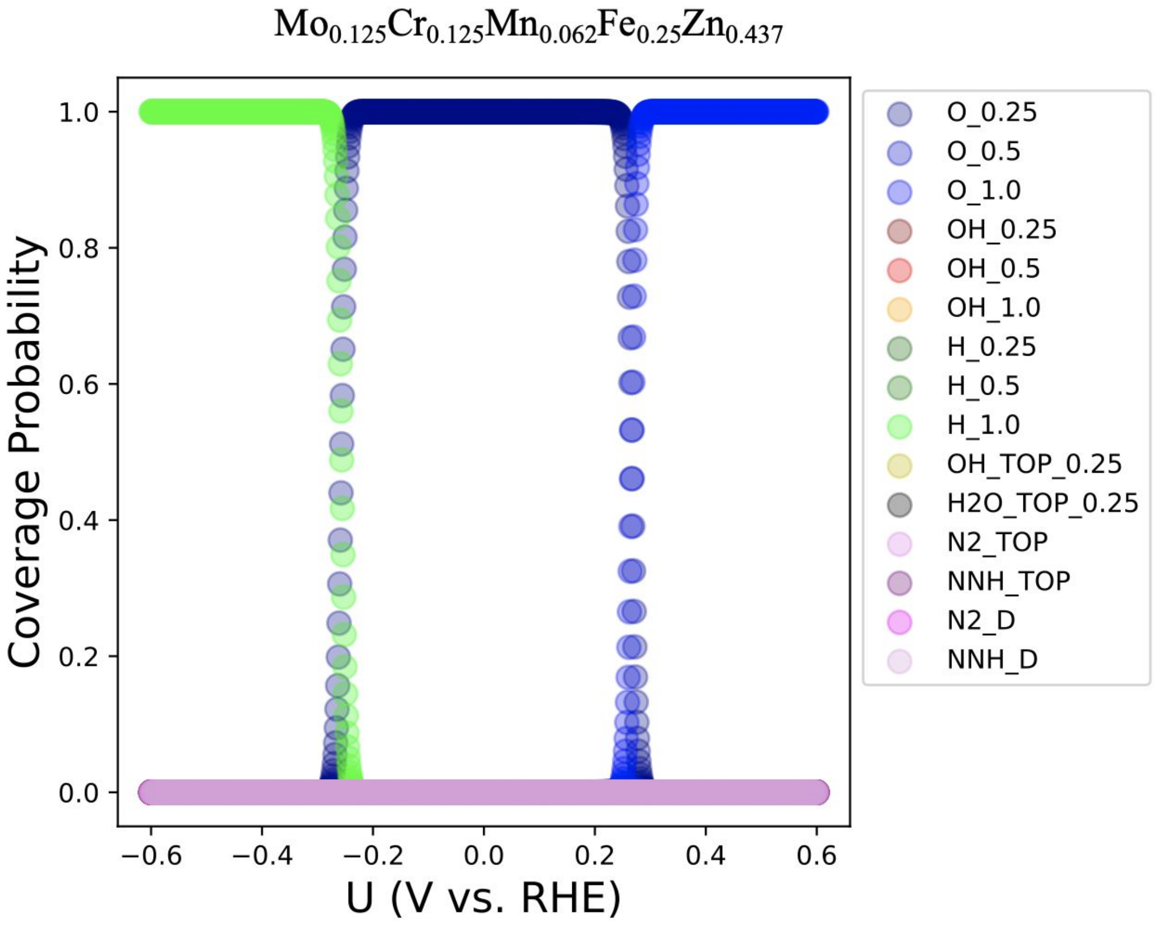

Figure S10: Computed surface coverages probabilities of the material $Mo_{0.125}Cr_{0.125}Mn_{0.062}Fe_{0.250}Zn_{0.437}$.

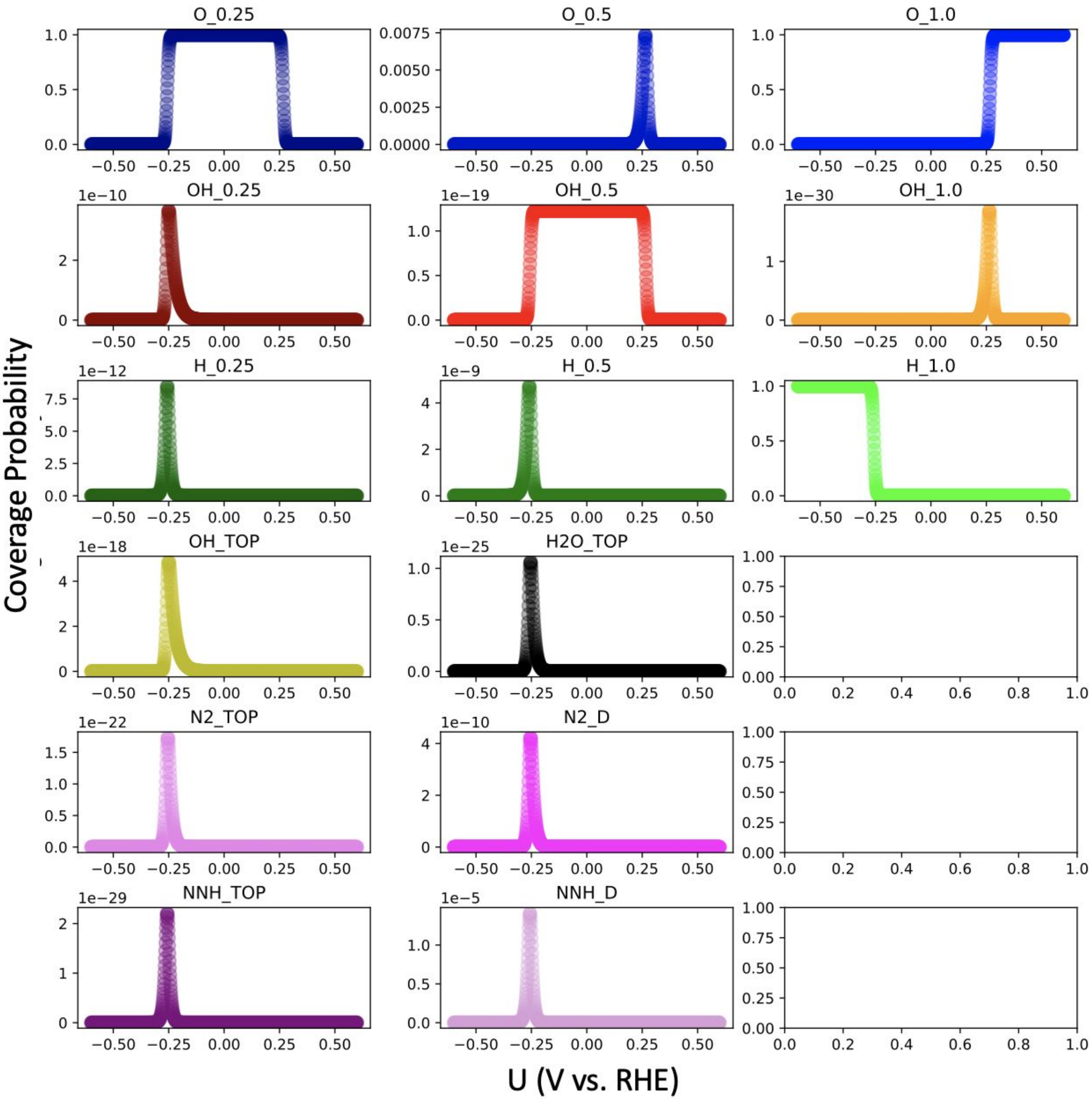

Figure S11: Computed surface coverages probabilities of the material $Mo_{0.125}Cr_{0.125}Mn_{0.062}Fe_{0.250}Zn_{0.437}$ and plotted separately for better visualization.

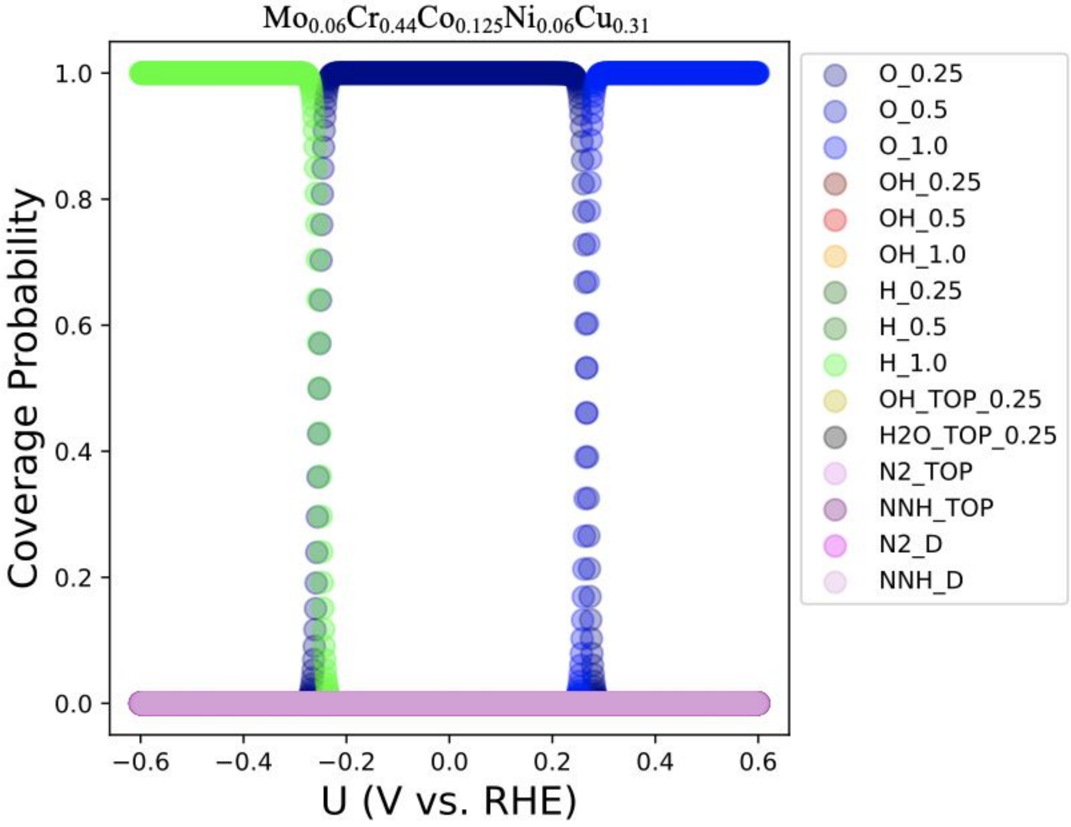

Figure S12: Computed surface coverages probabilities of the material $Mo_{0.06}Cr_{0.44}Co_{0.125}Ni_{0.06}Cu_{0.31}$.
